# Comparing Insulin Against Glucagon-Like Peptide-1 Receptor Agonists, Dipeptidyl Peptidase-4 Inhibitors, and Sodium-Glucose Cotransporter 2 Inhibitors on 5-Year Incident Heart Failure Risk for Patients With Type 2 Diabetes Mellitus: Real-World Evidence Study Using Insurance Claims

**DOI:** 10.2196/58137

**Published:** 2024-10-22

**Authors:** Xuan Wang, Anna M Plantinga, Xin Xiong, Sara J Cromer, Clara-Lea Bonzel, Vidul Panickan, Rui Duan, Jue Hou, Tianxi Cai

**Affiliations:** 1 Department of Population Health Sciences, University of Utah, Salt Lake City, UT, United States; 2 Department of Mathematics and Statistics, Williams College, Williamstown, MA, United States; 3 Department of Biostatistics, Harvard University, 677 Huntington Avenue, Boston, MA, 02115, United States, 1 617-432-4923; 4 Department of Medicine, Massachusetts General Hospital, Boston, MA, United States; 5 Department of Biomedical Informatics, Harvard University, Boston, MA, United States; 6 Department of Biostatistics, University of Minnesota, Minneapolis, MN, United States

**Keywords:** type 2 diabetes mellitus, diabetes, diabetes complications, heart failure, antidiabetic drug, diabetes pharmacotherapy, insulin, GLP-1 RA, DPP-4I, SGLT2I, real-world data, insurance data, claims data, glucagon-like peptide-1 receptor agonist, dipeptidyl peptidase-4 inhibitor, sodium-glucose cotransporter 2 inhibitor

## Abstract

**Background:**

Type 2 diabetes mellitus (T2DM) is a common health issue, with heart failure (HF) being a common and lethal long-term complication. Although insulin is widely used for the treatment of T2DM, evidence regarding the efficacy of insulin compared to noninsulin therapies on incident HF risk is missing among randomized controlled trials. Real-world evidence on insulin’s effect on long-term HF risk may supplement existing guidelines on the management of T2DM.

**Objective:**

This study aimed to compare insulin therapy against other medications on HF risk among patients with T2DM using real-world data extracted from insurance claims.

**Methods:**

A retrospective, observational study was conducted based on insurance claims data from a single health care network. The study period was from January 1, 2016, to August 11, 2021. The cohort was defined as patients having a T2DM diagnosis code. The inclusion criteria were patients who had at least 1 record of a glycated hemoglobin laboratory test result; full insurance for at least 1 year (either commercial or Medicare Part D); and received glucose-lowering therapy belonging to 1 of the following groups: insulin, glucagon-like peptide 1 receptor agonists (GLP-1 RAs), dipeptidyl peptidase-4 inhibitors (DPP-4Is), or sodium-glucose cotransporter-2 inhibitors (SGLT2Is). The main outcome was the 5-year incident HF rate. Baseline covariates, including demographic characteristics, comorbidities, and laboratory test results, were adjusted to correct for confounding.

**Results:**

After adjusting for a broad list of confounders, patients receiving insulin were found to be associated with an 11.8% (95% CI 11.0%‐12.7%), 12.0% (95% CI 11.5%‐12.4%), and 15.1% (95% CI 14.3%‐16.0%) higher 5-year HF rate compared to those using GLP-1 RAs, DPP-4Is, and SGLT2Is, respectively. Subgroup analysis showed that insulin’s effect of a higher HF rate was significant in the subgroup with high HF risk but not significant in the subgroup with low HF risk.

**Conclusions:**

This study generated real-world evidence on the association of insulin therapy with a higher 5-year HF rate compared to GLP-1 RAs, DPP-4Is, and SGLT2Is based on insurance claims data. These findings also demonstrated the value of real-world data for comparative effectiveness studies to complement established guidelines. On the other hand, the study shares the common limitations of observational studies. Even though high-dimensional confounders are adjusted, remaining confounding may exist and induce bias in the analysis.

## Introduction

Type 2 diabetes mellitus (T2DM) is a major health issue globally and particularly prevalent in the United States. More than 34 million US adults (13%) had diabetes as of 2018, of which 90% to 95% were T2DM, and complication from diabetes was the seventh leading cause of death in the United States in 2017 [1]. Cardiovascular disease (CVD) is a common and lethal complication of T2DM. Among patients with T2DM, the prevalence of heart failure (HF) is between 9% and 22%, which is 4 times higher than that for the general population [2]. Therefore, in addition to apparent short-term disease management goals such as glycemic control, it is important to evaluate the impact of T2DM therapies on long-term health outcomes such as incident HF [2-5].

Insulin therapy has a long history in the management of T2DM and remains one of the most effective and affordable treatments for glycemic control. Insulin is typically initiated after oral medications fail to control glycemia, but it is sometimes used in early-line treatments if a patient has contraindications for oral medications [6]. In recent years, next-generation medications guided by the analysis of disease pathways, such as glucagon-like peptide-1 receptor agonists (GLP-1 RAs), dipeptidyl peptidase-4 inhibitors (DPP-4Is), and sodium-glucose cotransporter 2 inhibitors (SGLT2Is), have become increasingly common alternatives or additions to insulin for second-line T2DM therapy.

Despite insulin’s frequent prescription for T2DM management, its impact on long-term incident HF risk has not been thoroughly assessed, especially in comparison to alternative therapy options [2,3]. A study of electronic medical records for nearly 10,000 patients with T2DM and age- and sex-matched controls found that insulin use was associated with a higher risk of both prevalent and incident congestive HF independent of glycemic control, confirming the importance of studying HF beyond successful glycemic control [7]. Several other large observational studies reported the association of insulin with increased risk of CVD-related outcomes, including comparisons across doses of insulin, of insulin monotherapy versus insulin plus metformin, and of insulin versus novel therapies (DPP-4Is or SGLT2Is grouped together) [8]. These studies did not cover all head-to-head comparisons between insulin and alternative therapies. Moreover, many of these studies adjusted for a somewhat limited set of covariates and comorbidities, raising concerns about bias from unmeasured confounding.

Recent evidence suggests that alternative second-line agents may lead to lower CVD risk profiles. For example, comparing the risk of macrovascular CVD outcomes between people with T2DM treated by insulin versus exenatide (a type of GLP-1 RA) in a large ambulatory care dataset, Paul et al [9] found that the risk of incident HF was significantly lower in the exenatide and exenatide+insulin groups compared to the insulin-only group. On the other hand, in a recent systematic review of cohort and nested case-control studies, Alkhezi et al [10] described conflicting evidence, with some studies showing a significantly lower risk of incident HF in GLP-1 RA groups compared to insulin groups and other studies showing no significant differences. Similar studies of SGLT2Is versus GLP-1 RAs and insulin versus DPP-4Is have found that SGLT2Is are associated with a lower risk of incident HF compared to GLP-1 RAs, and DPP-4Is have a significantly lower risk of CVD events than insulin in both a cohort without CVD and the general population (matched on propensity scores [PSs]) [11,12]. However, the outcome definition and inclusion criteria vary between studies, as do the baseline covariates included, making it difficult to directly compare results. Most of the existing studies are based on association studies, which lack causal interpretation. Additionally, insulin is included as a comparison group in very few studies, in part because of the difficulty of fully adjusting for confounding by indication. Patients receiving insulin therapy are generally different from those who are recommended for GLP-1 RAs, DPP-4Is, and SGLT2Is per current treatment guidelines. However, the optimal treatment option remains unclear for patients potentially receiving insulin or alternative therapies based on their predicted HF risks. Consequently, there is a pressing need for a more thorough and robust comparison of the risk of HF between insulin and alternative therapies, accounting carefully for confounding due to differences in patient populations, in order to guide T2DM management recommendations [13-18].

Randomized controlled trials (RCTs) are the gold standard for inferring causal differences in treatment effects, but because the study of HF outcomes requires a very long follow-up time and insulin is a generic medication, there is little incentive for private sponsors to support RCTs comparing insulin to patent-protected agents. Readily available real-world data (RWD), such as electronic health record data and insurance claims data, can be used to generate real-world evidence to fill in the blank. In contrast to highly regimented RCTs, real-world evidence on the therapy may offer both generalizable and personalized guidance for practice as supported by regulatory guidelines [19,20]. The much larger sample sizes and availability of data beyond restrictive RCT inclusion and exclusion criteria allow recommendations based on RWD to apply to a much broader population and to be tailored for specific subgroups. Therefore, our study aims to compare the effect of insulin against GLP-1 RAs, DPP-4Is, and SGLT2Is separately on long-term incident HF risk using real-world insurance claims data, applying a doubly robust estimation method to correct for potential confounding biases across a large set of baseline factors.

## Methods

### T2DM Data Source

We created the study data from the data factory inside the UnitedHealth Group Research and Development platform. The study period was from January 1, 2016, to August 11, 2021.

### Ethical Considerations

The UnitedHealth Group institutional review board approved the use of the insurance claims and electronic health record data for this study with a waiver of informed consent (RB20-1213: Precision Medicine of Type 2 Diabetes). Data have been deidentified.

### Data Curation and Feature Extraction

We identified T2DM diagnoses using a list of *International Classification of Diseases, Ninth Revision* (*ICD-9*) and *International Statistical Classification of Diseases, Tenth Revision* (*ICD-10*) codes related to T2DM and constructed the data mart from patients with these codes in the list. The date of the first T2DM diagnosis was identified. We excluded patients with likely type 1 diabetes mellitus. Inclusion criteria were at least 1 glycated hemoglobin (HbA_1c_) measurement through the study period (those with zero HbA_1c_ results were considered to have missing data); pharmacy claims for at least 1 of the 4 therapy mechanism groups of interest (insulin, GLP-1 RAs, DPP-4Is, and SGLT2Is); and insurance coverage classified as either “Commercial, Fully Insured (FI)” or “Medicare Advantage Part D (MAPD)” for at least 1 year. Individuals with an HF diagnosis prior to the first prescription of the therapy mechanism groups of interest were excluded. Data on demographics, other medications, laboratory test results, and comorbidities were also extracted for confounding adjustment.

**Figure 1.**
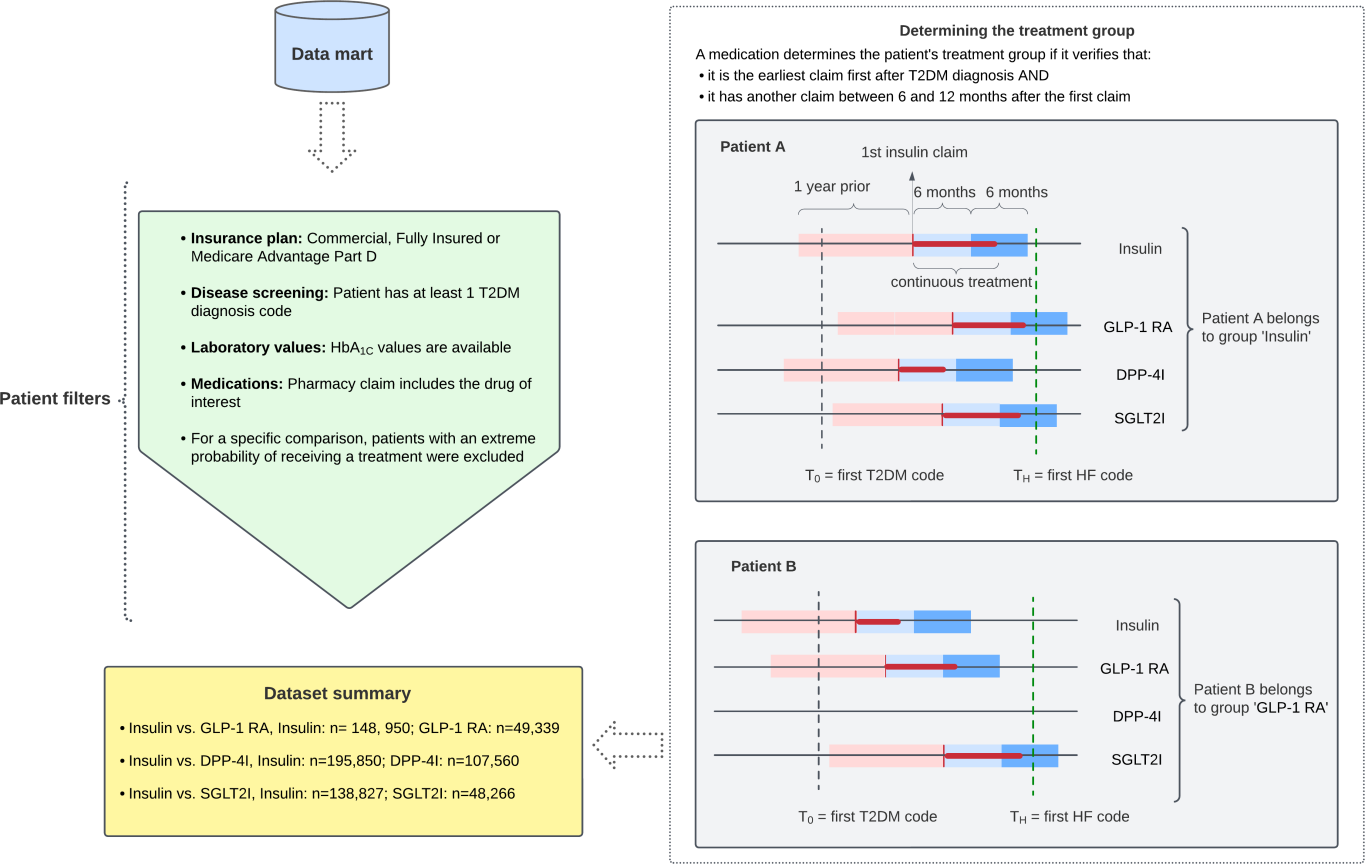
Schematic of data generation. Pink: 1-year window before prescription; light blue: 6-month window after prescription; blue: 12-month window after prescription. DDP-4I: dipeptidyl peptidase-4 inhibitor; GLP-1 RA: glucagon-like peptide 1 receptor agonist; HbA_1c_: glycated hemoglobin; HF: heart failure; SGLT2I: sodium-glucose cotransporter-2 inhibitor; T2DM: type 2 diabetes mellitus.

Patients were classified into treatment groups based on their earliest sustained medication among the 4 therapies in comparison. Because a quick switch from a recently prescribed therapy may represent a prolonged treatment decision process between the patient and provider, we required at least 2 insurance claims for the same therapy to indicate sustained use. Specifically, the medication received the earliest after study enrollment, with another claim between 6 and 12 months later, was identified as the patient’s treatment group for this study (Figure 1).

The outcome of interest was incident HF risk. We identified HF events by a list of *ICD-9* and *ICD-10* diagnosis codes mapped to PheWAS catalog codes (phecodes) [21]: 428.1, congestive HF nitric oxide synthases; 428.2, HF nitric oxide synthases; 428.3, HF with reduced ejection fraction (EF; systolic or combined HF); and 428.4, HF with preserved EF (diastolic HF). The observation time for each patient was the time from the first prescription date to either the first occurrence of an HF event or the last follow-up date, whichever was earlier. The median follow-up time was around 2.4 years. The overall follow-up time was around 5.4 years for insulin, 5.2 years for GLP-1 RAs, 5.4 years for DPP-4Is, and 5.4 years for SGLT2Is.

To account for confounding, we extracted demographic characteristics including age, sex, residential area (urban or rural), Medicaid coverage, socioeconomic index, and disease duration (from the T2DM diagnosis date to the first prescription date), as well as laboratory values for baseline HbA_1c_, high-density lipoprotein, low-density lipoprotein, and cholesterol up to 1 year prior to the first prescription date. Missing laboratory values were imputed by means. We accounted for confounding from the general health history, summarized by the high-dimensional counts of diagnosis codes in each phecode group during the year prior to treatment initiation [22]. Rare phecode features with less than 5% prevalence were excluded.

### Statistical Analysis

The analyses outlined in this section were conducted to compare the treatment effect of insulin versus GLP-1 RAs. The same analyses were repeated for insulin versus DPP-4Is and insulin versus SGLT2Is. We defined the 5-year average treatment effect (ATE) as the difference in HF-free rate (HF survival probability throughout the paper) between treatment groups at 5 years after the first prescription date. Because treatment decisions depend on baseline patient factors that are themselves associated with HF outcomes, adequate adjustment for these confounding biases is critical to infer treatment effects. We, therefore, applied a doubly robust estimation method involving 2 adjustments to account for treatment-by-indication biases, as visualized in Figure S1 in Multimedia Appendix 1. First, for the PS model, we used a logistic regression model with baseline covariates (demographics, laboratory results, diagnoses, and medication). We balanced baseline factors between treatment groups through the inverse probability of treatment weighting (IPW). Second, for the outcome regression (OR) model, we adjusted baseline factors in 2 Cox models (for insulin and GLP-1 RAs, respectively) to assess their association with HF risk. We used an adaptive LASSO (least absolute shrinkage and selection operator) penalized regression to fit both the PS and OR models [23], an approach that shrinks coefficients for uninformative covariates to zero and provides stable effect estimates for the informative covariates. In addition to the baseline covariates, the first 3 principal components of the comorbidities were added as covariates. Within each model, the associations with the covariate *age* can be nonlinear, and the associations with the other covariates are allowed to vary with age. Specifically, we included *g(age)* and the interaction of *g(age)* with other covariates, where *g* is an unknown function. We approximate *g(age)* by splines basis. In practice, we found that the commonly used b-spline or natural splines basis with 3 knots worked well. Equally spaced knots that cover most of the domain of the data for *age* were also desirable. That is, age was represented flexibly using basis splines with 3 equally spaced knots, and all models included the 3 age basis variables and their interactions with other covariates. This resulted in 3 sets of coefficient estimates in the model fitting results.

We also calculated covariate-specific ATEs (CATEs) to study ATEs among different subgroups: score-specific ATE. The score *S* was defined as the survival probability difference based on the 2 Cox models for the 2 treatment groups, where a positive score is in favor of insulin. The score can be considered as the personalized insulin prescription score. All analyses were performed using R software (R Foundation for Statistical Computing).

## Results

### Insulin Versus GLP-1 RAs

The baseline characteristics of eligible patients in the insulin and GLP-1 RA treatment groups are displayed in Table 1. For the insulin group, the mean age was 66.24 (SD 9.8) years, and 48.06% (71,579/148,950) were male, while for the GLP-1 RA group, the mean age was 62.44 (SD 11) years, and 43.91% (22,664/49,339) were male. As shown in Table 1 and Table S1 in Multimedia Appendix 1, covariate balance after IPW was much improved, with similar distributions of demographic characteristics, average laboratory values, and additional medications between the 2 groups after IPW.

**Figure 2.**
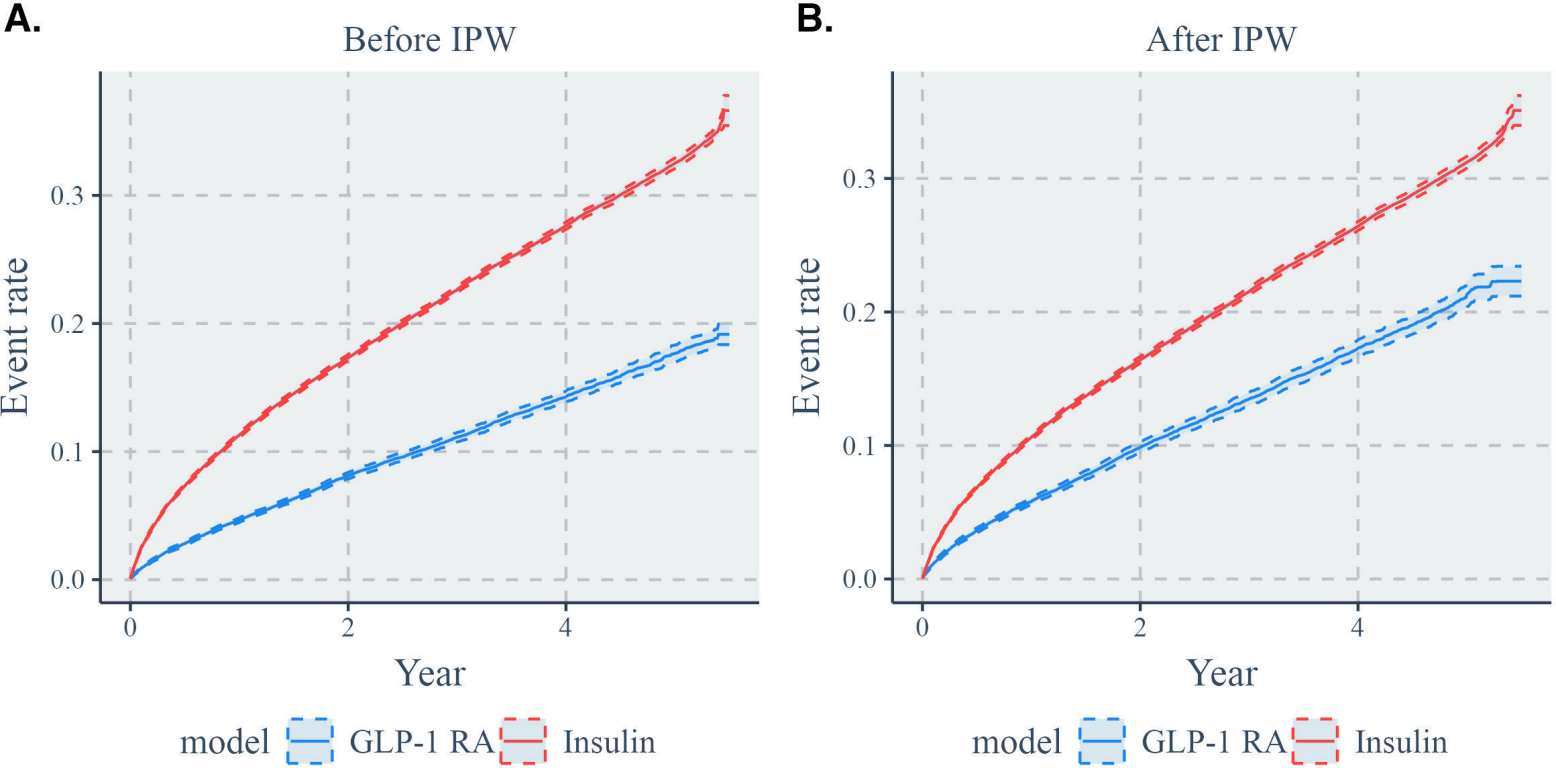
HF rates (A) before and (B) after IPW for the insulin versus GLP-1 RA comparison. GLP-1 RA: glucagon-like peptide 1 receptor agonist; HF: heart failure; IPW: inverse probability of treatment weighting.

The Kaplan-Meier estimates of HF probability across time, before and after balancing covariates through IPW, are visualized in Figure 2. These show that the HF rate for the insulin group was higher than that for the GLP-1 RA group.

Table 2 presents the doubly robust estimated 5-year HF rate for the 2 treatment groups under comparison, as well as the difference between the 2 rates (the estimated ATE). A positive ATE indicates that insulin group had a higher HF probability compared to the other treatment group. The estimated 5-year HF rate in the insulin group differed across the 3 comparisons due to differences in the populations’ distributions of baseline characteristics and the corresponding differences in HF risk, as well as the exclusion of some patients with an extreme probability of receiving a particular treatment due to their inability to be matched with the other treatment group.

**Table 1.** Baseline characteristics of patients included in the study (insulin vs GLP-1 RAs^a^).

Baseline characteristics	Before IPW^b^		After IPW	
	Insulin (n=148,950)	GLP-1 RAs (n=49,339)	Insulin (n=148,950)	GLP-1 RAs (n=49,339)
**Demographics**				
** Age at first prescription (y), mean (SD)**	66.24 (9.86)	62.44 (11)	65.39 (10.3)	64.79 (10.32)
** Disease duration (mo), mean (SD)**	2.67 (9.78)	7.45 (14.53)	3.55 (10.9)	4.97 (12.76)
** Male sex, n (%)**	71,579 (48.06)	21,662 (43.91)	70,594 (47.39)	22,664 (45.93)
** Medicaid coverage, n (%)**	1993 (1.34)	474 (0.96)	1923 (1.29)	580 (1.18)
**Rural status, n (%)**				
Rural	40,693 (27.32)	12,723 (25.79)	40,274 (27.04)	13,284 (26.92)
Urban	42,050 (28.23)	14,046 (28.47)	42,339 (28.43)	13,682 (27.73)
**Socioeconomic index, mean (SD)**	51.99 (2.95)	52.19 (3.01)	52.01 (2.95)	52.16 (3.01)
**Laboratory values, mean (SD)**				
HbA_1c_^c^ (%)	8.52 (1.1)	8.25 (1.31)	8.5 (1.14)	8.38 (1.26)
Cholesterol (mg/dL)	170.87 (22.35)	170.91 (26.83)	170.86 (23.29)	170.86 (24.92)
HDL^d^ (mg/dL)	45.51 (6.35)	45.46 (7.82)	45.46 (6.64)	45.48 (7.15)
LDL^e^ (mg/dL)	90.04 (17.51)	90.17 (21.73)	90.02 (18.26)	90.1 (20.12)
**Additional medications, mean (SD)**				
Metformin	0.6 (0.82)	0.98 (0.95)	0.69 (0.88)	0.76 (0.88)
Statins	0.73 (0.84)	0.97 (0.94)	0.79 (0.88)	0.84 (0.88)
Sulfonylureas	0.32 (0.68)	0.5 (0.86)	0.36 (0.74)	0.41 (0.77)
Thiazolidinediones	0.07 (0.34)	0.13 (0.47)	0.09 (0.38)	0.1 (0.4)
**Other characteristics, mean (SD)**				
PC1^f^	−0.02 (2.29)	0.02 (2.22)	−0.02 (2.35)	0.01 (2.21)
PC2^g^	0.1 (0.83)	0.45 (0.92)	0.19 (0.88)	0.25 (0.87)
PC3^h^	0 (0.8)	0 (1)	0 (0.85)	0 (0.9)
S^i^	−0.13 (0.08)	−0.1 (0.08)	−0.13 (0.08)	−0.12 (0.08)

a GLP-1 RA: glucagon-like peptide 1 receptor agonist.

b IPW: inverse probability of treatment weighting.

c HbA_1c_: glycated hemoglobin.

d HDL: high-density lipoprotein.

e LDL: low-density lipoprotein.

f PC1: the first principal component of the comorbidities.

g PC2: the second principal component of the comorbidities.

h PC3: the third principal component of the comorbidities.

i S: model-based survival probability difference.

**Table 2.** Doubly robust estimates and SE of heart failure (HF) rates for each treatment group and the difference between HF rates (ATE^a^).

Comparison	Insulin group, estimate (SE)	Treatment group, estimate (SE)	Difference (SE)
Insulin vs GLP-1 RA^b^	0.293 (0.002)	0.175 (0.004)	0.118 (0.004)
Insulin vs DPP-4I^c^	0.348 (0.002)	0.229 (0.002)	0.120 (0.002)
Insulin vs SGLT2I^d^	0.281 (0.002)	0.130 (0.004)	0.151 (0.005)

a ATE: average treatment effect.

b GLP-1 RA: glucagon-like peptide 1 receptor agonist.

c DDP-4I: dipeptidyl peptidase-4 inhibitor.

d SGLT2I: sodium-glucose cotransporter-2 inhibitor.

Focusing first on the comparison between insulin and GLP-1 RAs, patients receiving insulin had a higher 5-year HF rate of 29.3% (95% CI 28.9%‐29.7%) compared to patients receiving GLP-1 RAs, whose estimated 5-year HF rate was 17.5% (95% CI 16.8%‐18.2%). Therefore, the estimate of the ATE was 11.8% (95% CI 11.7%-12.7%), suggesting that the probability of having HF 5 years after treatment initiation was nearly 12% higher for patients receiving insulin compared to those receiving GLP-1 RAs.

**Figure 3.**
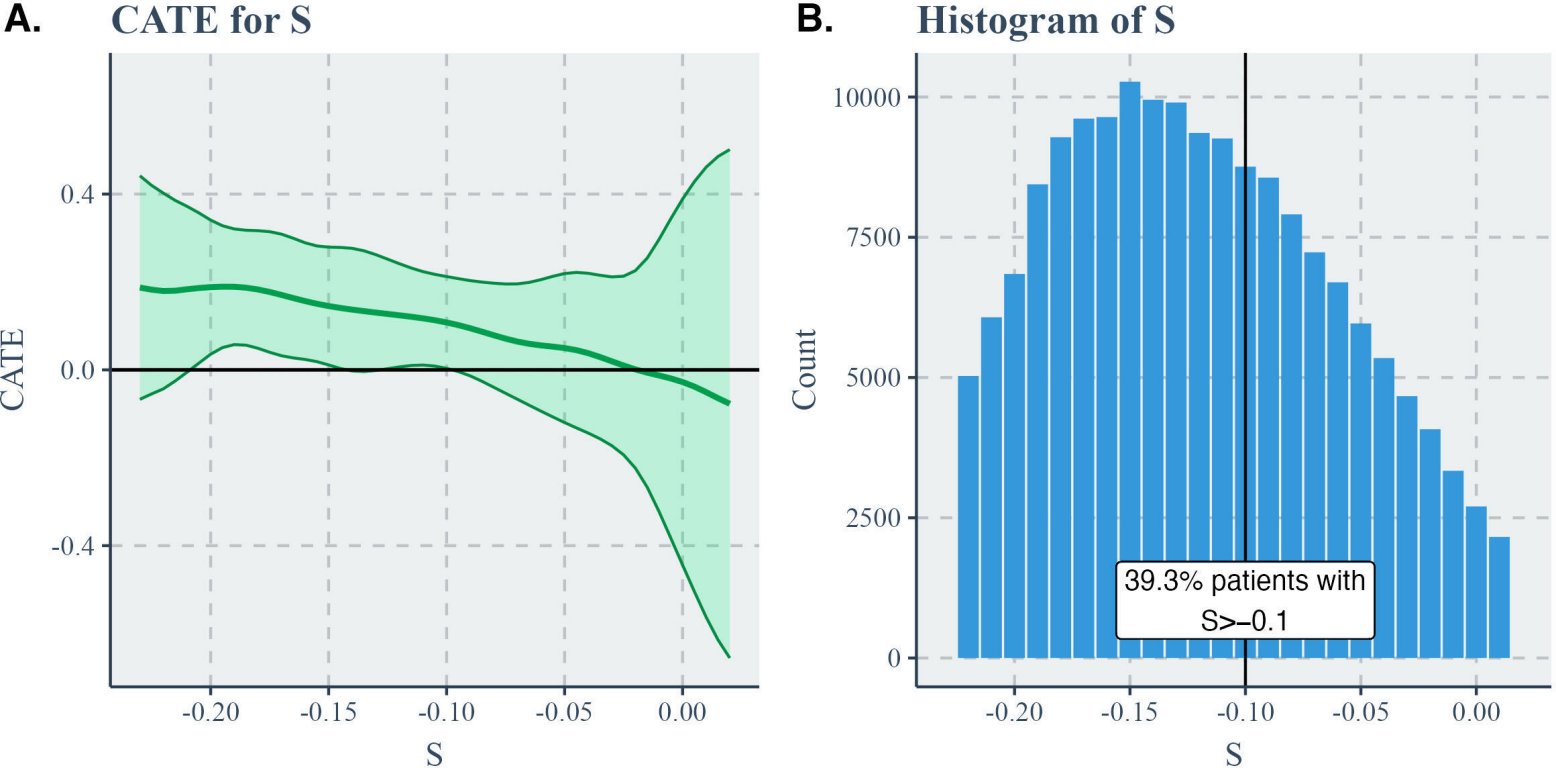
CATE estimates for the insulin versus GLP-1 RA comparison: (A) CATE for S and (B) histogram of S. CATE: covariate-specific average treatment effect; GLP-1 RA: glucagon-like peptide 1 receptor agonist; S: model-based survival probability difference.

CATEs, shown in Figure 3, can provide additional detail. The score-specific ATE was significantly positive when the score *S* (model-based survival probability difference) was positive but was no longer significant when *S* was negative. Thus, the score *S* is a relatively good indicator of whether insulin is better than GLP-1 RAs for a patient in consideration of their long-term heart health. Since most patients had positive *S* scores, the score-specific ATE supports the other estimates in suggesting that GLP-1 RAs were related to lower HF risk for most patients. In addition, we identified a substantial subset (nonpositive score) on which insulin had noninferiority compared to GLP-1 RAs.

In examining the baseline features selected by the adaptive LASSO procedure to be included in the PS and OR models, we observed confounders with clinical relevance to both treatment assignment and HF outcomes (Table S2 in Multimedia Appendix 1). Covariates associated with treatment assignment were indicated by a nonzero coefficient in the PS model, including age, Medicaid insurance, HbA_1c_ levels, additional medications taken, diabetic retinopathy, electrolyte imbalance, and several other baseline covariates. Fewer baseline covariates were selected in the Cox models for HF outcomes, including demographics, such as Medicaid insurance, and disease diagnoses, such as atrial fibrillation and coronary atherosclerosis. Many of the LASSO-selected features were associated with the treatment group but not HF outcomes, indicated by a nonzero PS coefficient but zero Cox coefficients, or with HF outcomes but not the treatment group, indicated by a zero PS coefficient but nonzero Cox coefficient(s).

### Insulin Versus DPP-4Is

Results were broadly similar for the insulin versus DPP-4I comparison. Baseline characteristics are shown Table S3 in Multimedia Appendix 1, and again, covariate balance between the treatment groups was much improved after IPW. The estimated ATE was nearly identical, at 12.0% (95% CI 11.5%-12.4%), although the estimated HF rates were higher in this population than in the previous comparison. In this case, the estimated 5-year HF rate for patients receiving insulin was 34.8% (95% CI 34.5%‐35.2%), whereas the rate for those receiving DPP-4Is was 22.9% (95% CI 22.5%‐23.3%; Figure S2 in Multimedia Appendix 1). The differences in the estimated 5-year HF rate between this analysis and the insulin versus GLP-1 RA analysis were likely due to differences in baseline characteristics. For example, the after-IPW frequency of comorbidities such as chronic renal failure, coronary atherosclerosis, and hypertensive heart or renal disease were all higher in the insulin versus DPP-4I comparison, and the latter was also associated with older age and a higher proportion of Medicaid coverage.

As in the previous comparison, the score-specific ATE (Figure S3 in Multimedia Appendix 1) again suggests that the score could be a useful indicator of optimal treatment, but that in most cases, the insulin group had a higher HF rate than the DPP-4I group. The LASSO-selected coefficients for treatment assignment (PS model) and HF time (Cox model) also included many of the same baseline factors as above (Table S4 in Multimedia Appendix 1).

### Insulin Versus SGLT2Is

For the comparison of insulin and SGLT2Is, the estimated ATE was larger, at 15.1% (95% CI 14.3% to 16.0%; Table 2). The 5-year HF rates were also lower than in the prior 2 comparisons (insulin versus GLP-1 RAs and insulin versus DPP-4Is), with the estimated 5-year HF rate reaching 28.1% (95% CI 27.7%‐28.5%) in the insulin group and 13.0% (95% CI 12.3%‐13.7%) in the SGLT2I group (Figure S4 in Multimedia Appendix 1). Baseline characteristics after IPW for this comparison were similar to those for the insulin versus GLP-1 RA comparison (Table S5 in Multimedia Appendix 1), so the comparable HF rates between the 2 analyses were to be expected. The score-specific ATE (Figure S5 in Multimedia Appendix 1) and selected covariates (Table S6 in Multimedia Appendix 1) were also similar to the prior 2 comparisons.

## Discussion

### Principal Findings

We used an intention-to-treat strategy for medication assignment by assigning patients to the treatment group corresponding to the medication they received the earliest after study enrollment, with another claim between 6 and 12 months later. With more than 1 sustained medication, we defined treatment group based on the earliest one using the intention-to-treat strategy. In practice, with more than 1 sustained medication, it is hard to distinguish these medications as we do not know which medication takes the main effect. It would be better to include additional exclusion strategies that can reduce bias in assigning treatment groups under the current strategy. On the other hand, future analyses could incorporate additional information about patients switching between treatments over the course of the study, or patients taking multiple study medications simultaneously. As clinical guidelines continue to be clarified, this additional information could also aid in defining relevant medication grouping strategies.

The outcome definition is heterogeneous, which is both a strength and a weakness of the study design. Although HF with reduced EF (code 428.3) and HF with preserved EF (code 428.4) are clinically different in terms of risk factors, prognosis, and treatment [24], we aimed to capture all cardiac-related events through the generic outcome definition. Further research is needed to clarify distinct associations with these 2 diseases.

Further, the use of insurance claims data is limited by data that may be incomplete, especially in those with limited access to health care or who disenroll from the studied insurance plan. For example, the inferred disease duration may not be an accurate measure as people may have received their diagnosis either before the first available data in the system or before they enrolled in this insurance plan. Similarly, because the first prescription date was defined as the earliest prescription after study enrollment, it is possible that patients with “first prescriptions” in the first few months of the study actually started that therapy prior to the study start date, resulting in a longer time to HF than we observed. These covariates curated 1 year prior to the first prescription are also potentially biased due to the potential for an early first prescription (participants enrolled later in the study would have the full year, whereas participants with the first prescription between January 1, 2016, and January 1, 2017, would have proportionally fewer phecode occurrences).

Also, the restriction to those with commercial or Medicare Part D insurance, although necessary to minimize the chance that patients are filling medication prescriptions through another insurance provider, imposes some selection bias. Most Medicare beneficiaries (74.4%) have Part D coverage, and most of the remainder have drug coverage through other providers (16.3% of beneficiaries), but 9.1% of beneficiaries had no drug coverage at all as of 2019. Those with private coverage tended to have higher income, were less likely to be eligible through disability, and were more likely to have attended college compared to other groups; those without drug coverage had characteristics that were between those covered by Part D and those with alternative drug coverage [25].

Although we adjusted extensively for potential confounding effects, the use of real-world (and therefore observational) data still results in the possibility of residual confounding, in particular confounding by indication for the use of insulin, since insulin users are generally “sicker” in many ways than noninsulin users.

By comparing insulin to each of the alternative medications separately, our study aimed to maximize the balance between the treatment groups with respect to baseline covariates using PS modeling and IPW. Studies comparing HF rates among more than 2 therapies can be done with a multinomial regression for the PS model, which is warranted to further guide treatment recommendations.

### Conclusion

In this study using real-world, insurance claims data, we compared insulin to other second-line T2DM medications (GLP-1 RAs, DPP-4Is, and SGLT2Is) with respect to incident HF risk. We used a doubly robust, augmented IPW estimation that extensively adjusted for high-dimensional confounding factors in both the PS and OR models, using a data-driven approach for feature selection implemented through a LASSO sparsity penalty in each model. While the results presented above were based on the primary outcome of 5-year HF rate, the results were very similar for 4-year HF rate.

We found that patients with T2DM treated with insulin have a significantly higher risk of 5-year incident HF compared with each of the 3 alternative treatments—GLP-1 RAs, DPP-4Is, and SGLT2Is. The score *S* (model-based survival probability difference) is associated with ATE and may provide treatment guidance. By using RWD with a large sample size and adjusting for a large set of possible confounders using doubly robust estimation, our approach provides potentially clinically applicable estimates of treatment effects on HF rates, particularly in the absence of clinical trial data. The largest difference in HF rates was between insulin and SGLT2Is. This is consistent with prior studies, which broadly conclude that SGLT2Is are associated with reduced rates of HF compared to placebo or other treatments. However, as prior studies have generally been based on association studies and restricted populations (as in secondary analyses of clinical trials) or have adjusted for fewer potential confounders, our results strengthen and generalize these conclusions.

## Supplementary material

10.2196/58137 Multimedia Appendix 1 Doubly robust estimation scheme, baseline characteristics of patients, variables selected by the propensity score and outcome regression models, and additional results for dipeptidyl peptidase-4 inhibitors and sodium-glucose cotransporter-2 inhibitors.

## Abbreviations

ATE: average treatment effect
CATE: covariate-specific average treatment effect
CVD: cardiovascular disease
DDP-4I: dipeptidyl peptidase-4 inhibitor
EF: ejection fraction
GLP-1 RA: glucagon-like peptide 1 receptor agonist
HbA_1c_: glycated hemoglobin
HF: heart failure
*ICD-10*: *International Statistical Classification of Diseases, Tenth Revision*
*ICD-9*: *International Classification of Diseases, Ninth Revision*
IPW: inverse probability of treatment weighting
LASSO: least absolute shrinkage and selection operator
OR: outcome regression
PS: propensity score
RCT: randomized controlled trial
RWD: real-world data
SGLT2I: sodium-glucose cotransporter-2 inhibitor
T2DM: type 2 diabetes mellitus
