# Supplementary material for: Comparing Insulin Against Glucagon-Like Peptide-1 Receptor Agonists, Dipeptidyl Peptidase-4 Inhibitors, and Sodium-Glucose Cotransporter 2 Inhibitors on 5-Year Incident Heart Failure Risk for Patients With Type 2 Diabetes Mellitus: Real-World Evidence Study Using Insurance Claims
Source: JMIR Diabetes. 2024 Oct 22;9:e58137. doi: 10.2196/58137 (PMC11520261; doi:10.2196/58137)

**Multimedia Appendix 1**

### Figure S1. Scheme of the doubly robust estimation of ATE.


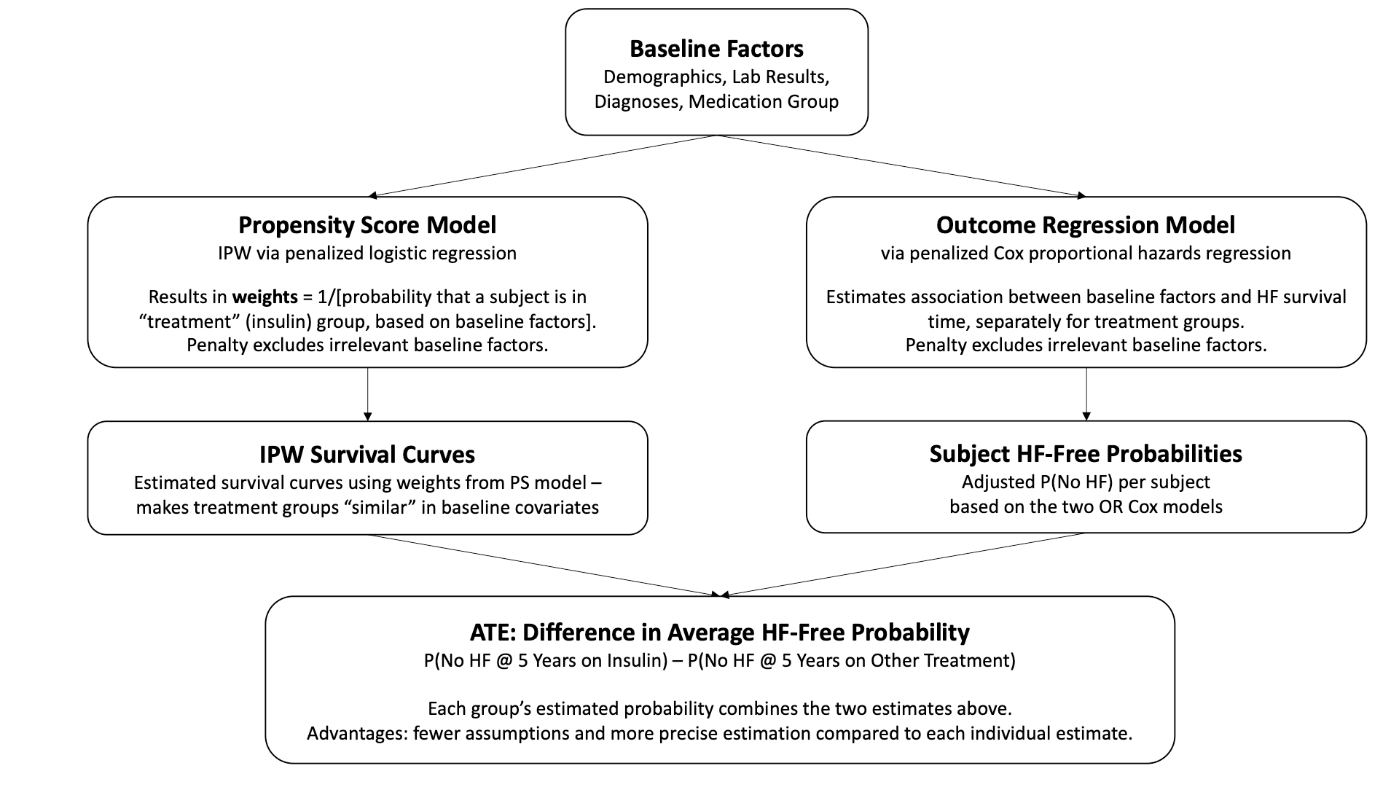


### Table S1. Baseline characteristics of patients included in the study.

Values reported are Mean (SD) or N (%). N(Insulin): 148,950; N(GLP-1): 49,339.

| **Disease** | Before IPW | | After IPW | |
| --- | --- | --- | --- | --- |
|  | Insulin | GLP1 | Insulin | GLP1 |
| Abnormal electrocardiogram [ECG] [EKG] | 0.06 (0.29) | 0.07 (0.33) | 0.06 (0.3) | 0.07 (0.31) |
| Abnormal glucose | 0.07 (0.32) | 0.12 (0.42) | 0.08 (0.35) | 0.09 (0.36) |
| Abnormality of gait | 0.05 (0.31) | 0.05 (0.34) | 0.05 (0.32) | 0.05 (0.32) |
| Acute renal failure | 0.06 (0.34) | 0.05 (0.34) | 0.06 (0.34) | 0.06 (0.36) |
| Anxiety disorder | 0.14 (0.51) | 0.21 (0.63) | 0.16 (0.55) | 0.17 (0.57) |
| Atrial fibrillation and flutter | 0.08 (0.43) | 0.08 (0.46) | 0.08 (0.44) | 0.08 (0.46) |
| Cerebral ischemia | 0.04 (0.28) | 0.04 (0.29) | 0.05 (0.29) | 0.04 (0.29) |
| Chronic pain | 0.08 (0.36) | 0.1 (0.42) | 0.08 (0.38) | 0.09 (0.39) |
| Chronic renal failure [CKD] | 0.19 (0.62) | 0.17 (0.59) | 0.19 (0.62) | 0.19 (0.62) |
| Coronary atherosclerosis | 0.24 (0.72) | 0.24 (0.73) | 0.24 (0.73) | 0.24 (0.73) |
| Cough | 0.11 (0.4) | 0.16 (0.48) | 0.12 (0.42) | 0.14 (0.44) |
| Degeneration of intervertebral disc | 0.08 (0.39) | 0.12 (0.48) | 0.09 (0.41) | 0.1 (0.43) |
| Depression | 0.17 (0.57) | 0.24 (0.68) | 0.19 (0.6) | 0.2 (0.62) |
| Dermatophytosis | 0.09 (0.35) | 0.1 (0.37) | 0.09 (0.36) | 0.09 (0.37) |
| Diabetic retinopathy | 0.15 (0.5) | 0.12 (0.48) | 0.14 (0.5) | 0.14 (0.52) |
| Dizziness and giddiness | 0.07 (0.33) | 0.09 (0.39) | 0.07 (0.34) | 0.07 (0.35) |
| Edema | 0.08 (0.34) | 0.09 (0.38) | 0.08 (0.36) | 0.08 (0.37) |
| Electrolyte imbalance | 0.08 (0.37) | 0.08 (0.38) | 0.08 (0.37) | 0.08 (0.38) |
| Esophagitis, GERD and related diseases | 0.2 (0.59) | 0.28 (0.69) | 0.22 (0.62) | 0.24 (0.64) |
| Essential hypertension | 1.13 (1.2) | 1.43 (1.24) | 1.21 (1.23) | 1.27 (1.22) |
| Hyperlipidemia | 0.95 (1.09) | 1.29 (1.14) | 1.04 (1.12) | 1.1 (1.11) |
| Hypertensive heart and/or renal disease | 0.15 (0.52) | 0.15 (0.54) | 0.15 (0.53) | 0.15 (0.54) |
| Hypothyroidism NOS | 0.19 (0.6) | 0.29 (0.74) | 0.22 (0.64) | 0.24 (0.67) |
| Insomnia | 0.05 (0.29) | 0.08 (0.36) | 0.06 (0.31) | 0.07 (0.33) |
| Muscle weakness | 0.05 (0.34) | 0.06 (0.39) | 0.05 (0.35) | 0.05 (0.36) |
| Obesity | 0.44 (0.87) | 0.71 (1.03) | 0.5 (0.92) | 0.56 (0.94) |
| Open-angle glaucoma | 0.05 (0.28) | 0.07 (0.33) | 0.06 (0.29) | 0.06 (0.31) |
| Osteoarthritis; localized | 0.17 (0.57) | 0.25 (0.7) | 0.19 (0.6) | 0.21 (0.64) |
| Osteoarthrosis NOS | 0.07 (0.34) | 0.09 (0.38) | 0.08 (0.35) | 0.08 (0.36) |
| Other abnormal blood chemistry | 0.05 (0.28) | 0.08 (0.34) | 0.06 (0.29) | 0.06 (0.3) |
| Other chronic nonalcoholic liver disease | 0.07 (0.36) | 0.1 (0.42) | 0.08 (0.38) | 0.08 (0.37) |
| Other dyspnea | 0.07 (0.32) | 0.09 (0.37) | 0.07 (0.34) | 0.08 (0.35) |
| Peripheral vascular disease, unspecified | 0.06 (0.31) | 0.05 (0.31) | 0.06 (0.31) | 0.06 (0.32) |
| Senile cataract | 0.14 (0.45) | 0.17 (0.5) | 0.15 (0.46) | 0.16 (0.48) |
| Shortness of breath | 0.13 (0.47) | 0.16 (0.52) | 0.14 (0.48) | 0.15 (0.5) |
| Sleep apnea | 0.21 (0.66) | 0.34 (0.82) | 0.24 (0.71) | 0.27 (0.73) |
| Spondylosis without myelopathy | 0.1 (0.44) | 0.14 (0.53) | 0.11 (0.47) | 0.12 (0.49) |
| Type 1 diabetes | 0.05 (0.24) | 0.03 (0.2) | 0.04 (0.24) | 0.04 (0.23) |
| Type 2 diabetes | 1.71 (1.39) | 2.11 (1.28) | 1.82 (1.4) | 1.89 (1.32) |
| Vitamin D deficiency | 0.18 (0.52) | 0.3 (0.66) | 0.21 (0.56) | 0.23 (0.58) |

### **Table S2.** Variables selected by PS and OR models. Each block of columns is one set of coefficients for each age spline basis. “cox1” indicates the outcome regression Cox model for insulin and “cox0” indicates the outcome regression Cox model for the comparison treatment group (GLP-1). Coefficient estimates significantly different from zero are colored red.

|  | 1 | | | | | | 2 | | | | | | 3 | | | | | |
| --- | --- | --- | --- | --- | --- | --- | --- | --- | --- | --- | --- | --- | --- | --- | --- | --- | --- | --- |
|  | propen | propen pval | cox1 | cox1 pval | cox0 | cox0 pval | propen | propen pval | cox1 | cox1 pval | cox0 | cox0 pval | propen | propen pval | cox1 | cox1 pval | cox0 | cox0 pval |
| **Demographics** |  |  |  |  |  |  |  |  |  |  |  |  |  |  |  |  |  |  |
| Age at First Prescription | 0.08 | 0.07 | 0.03 | 0.03 | 0.02 | 0.10 | -0.65 | 0.01 | 0.00 | #N/A | 0.00 | #N/A | 0.89 | 0.01 | 0.00 | #N/A | 0.04 | 0.10 |
| Disease Duration (months) | 0.00 | 0.32 | 0.00 | 0.02 | 0.00 | 0.19 | -0.03 | 0.00 | 0.00 | 0.08 | 0.00 | 0.25 | 0.01 | 0.05 | 0.00 | 0.12 | 0.00 | 0.32 |
| Male | 0.02 | 0.37 | 0.04 | 0.01 | 0.08 | 0.01 | 0.16 | 0.00 | 0.00 | 0.32 | 0.00 | #N/A | -0.33 | 0.00 | 0.00 | 0.20 | 0.00 | #N/A |
| Medicaid | -0.09 | 0.56 | 0.15 | 0.01 | 0.21 | 0.02 | 0.67 | 0.00 | 0.09 | 0.04 | 0.00 | #N/A | -0.75 | 0.00 | -0.02 | 0.24 | -0.15 | 0.06 |
| Rural | 0.02 | 0.62 | 0.04 | 0.00 | 0.09 | 0.01 | 0.07 | 0.09 | 0.03 | 0.00 | 0.02 | 0.15 | 0.04 | 0.51 | -0.02 | 0.04 | -0.04 | 0.19 |
| Socioeconomic Index | 0.00 | #N/A | 0.00 | #N/A | 0.00 | 0.13 | 0.00 | #N/A | 0.00 | 0.03 | 0.00 | 0.17 | 0.00 | #N/A | 0.01 | 0.00 | 0.01 | 0.00 |
| Urban | 0.10 | 0.00 | 0.00 | 0.69 | 0.01 | 0.16 | -0.01 | 0.81 | -0.02 | 0.05 | 0.00 | #N/A | 0.06 | 0.12 | 0.08 | 0.00 | 0.10 | 0.10 |
| **Laboratory** |  |  |  |  |  |  |  |  |  |  |  |  |  |  |  |  |  |  |
| A1C | 0.06 | 0.00 | 0.01 | 0.00 | 0.00 | 0.03 | 0.31 | 0.00 | 0.00 | #N/A | 0.00 | #N/A | -0.05 | 0.18 | 0.00 | 0.06 | 0.00 | 0.32 |
| Cholesterol | 0.00 | #N/A | 0.00 | 0.06 | 0.00 | 0.18 | 0.00 | #N/A | 0.00 | 0.32 | 0.00 | 0.25 | 0.00 | #N/A | 0.00 | 0.09 | 0.00 | 0.09 |
| HDL | 0.00 | #N/A | 0.00 | 0.17 | 0.00 | 0.32 | 0.00 | #N/A | 0.00 | 0.16 | 0.00 | 0.32 | 0.00 | #N/A | 0.00 | 0.01 | 0.00 | 0.02 |
| LDL | 0.00 | #N/A | 0.00 | 0.02 | 0.00 | 0.02 | 0.00 | #N/A | 0.00 | #N/A | 0.00 | 0.32 | 0.00 | #N/A | 0.00 | 0.02 | 0.00 | 0.05 |
| **Additional Medications** |  |  |  |  |  |  |  |  |  |  |  |  |  |  |  |  |  |  |
| Metformin | 0.00 | 0.99 | 0.00 | 0.32 | 0.00 | 0.32 | -0.30 | 0.00 | -0.01 | 0.27 | 0.00 | #N/A | 0.27 | 0.00 | 0.01 | 0.21 | 0.00 | 0.24 |
| Statins | -0.02 | 0.46 | 0.00 | 0.32 | 0.00 | 0.32 | -0.04 | 0.20 | 0.00 | 0.28 | 0.00 | #N/A | 0.16 | 0.01 | 0.00 | 0.98 | 0.01 | 0.16 |
| Sulfonylureas | -0.06 | 0.03 | 0.00 | 0.30 | 0.00 | #N/A | -0.09 | 0.04 | 0.00 | 0.32 | 0.00 | #N/A | 0.09 | 0.11 | 0.00 | 0.32 | -0.01 | 0.32 |
| Thiazolidinediones | -0.02 | 0.65 | 0.00 | 0.79 | -0.02 | 0.25 | -0.47 | 0.00 | 0.00 | 0.09 | 0.00 | #N/A | -0.18 | 0.12 | 0.05 | 0.02 | 0.02 | 0.26 |
| **Others** |  |  |  |  |  |  |  |  |  |  |  |  |  |  |  |  |  |  |
| PC1 | 0.00 | 0.99 | 0.00 | 0.89 | 0.00 | 0.41 | 0.02 | 0.39 | 0.00 | #N/A | 0.00 | #N/A | -0.06 | 0.64 | 0.00 | #N/A | 0.00 | 0.32 |
| PC2 | -0.02 | 0.14 | -0.03 | 0.00 | -0.02 | 0.06 | -0.23 | 0.00 | -0.56 | 0.00 | -0.44 | 0.00 | 0.07 | 0.04 | 0.06 | 0.00 | 0.08 | 0.01 |
| PC3 | 0.01 | 0.63 | 0.00 | 0.87 | 0.01 | 0.43 | -0.01 | 0.84 | 0.00 | #N/A | 0.00 | 0.32 | 0.03 | 0.20 | 0.01 | 0.38 | -0.01 | 0.17 |
| S | -2.89 | 0.00 | -2.11 | 0.00 | -0.51 | 0.00 | 0.01 | 0.32 | -5.80 | 0.00 | 1.22 | 0.02 | -0.18 | 0.29 | 0.00 | 0.32 | -0.25 | 0.21 |
| **Disease** |  |  |  |  |  |  |  |  |  |  |  |  |  |  |  |  |  |  |
| Abnormal electrocardiogram | 0.10 | 0.11 | 0.02 | 0.18 | -0.01 | 0.62 | -0.09 | 0.16 | 0.02 | 0.07 | 0.01 | 0.32 | -0.19 | 0.01 | -0.04 | 0.10 | 0.00 | 0.80 |
| Abnormal glucose | 0.09 | 0.13 | 0.00 | 0.54 | -0.01 | 0.16 | -0.21 | 0.01 | -0.01 | 0.14 | 0.00 | #N/A | 0.23 | 0.04 | 0.05 | 0.04 | 0.01 | 0.16 |
| Abnormality of gait | 0.05 | 0.56 | 0.01 | 0.17 | -0.01 | 0.14 | 0.06 | 0.49 | 0.00 | 0.76 | 0.00 | 0.32 | -0.12 | 0.15 | 0.01 | 0.79 | 0.02 | 0.21 |
| Acute renal failure | -0.01 | 0.79 | 0.00 | 0.70 | 0.03 | 0.16 | 0.23 | 0.00 | 0.03 | 0.10 | 0.00 | 0.32 | -0.09 | 0.35 | -0.01 | 0.32 | -0.08 | 0.10 |
| Anxiety disorder | 0.04 | 0.21 | 0.01 | 0.29 | 0.01 | 0.14 | -0.01 | 0.83 | 0.00 | 0.32 | 0.00 | #N/A | 0.01 | 0.82 | 0.01 | 0.18 | 0.00 | #N/A |
| Atrial fibrillation and flutter | -0.04 | 0.41 | 0.13 | 0.00 | 0.12 | 0.00 | -0.02 | 0.71 | 0.29 | 0.00 | 0.31 | 0.00 | -0.02 | 0.55 | -0.06 | 0.01 | -0.03 | 0.19 |
| Cerebral ischemia | 0.06 | 0.18 | 0.01 | 0.21 | 0.00 | 0.20 | 0.14 | 0.01 | 0.01 | 0.12 | 0.03 | 0.10 | -0.18 | 0.10 | 0.00 | 0.66 | -0.04 | 0.25 |
| Chronic pain | 0.05 | 0.12 | 0.00 | 0.32 | 0.01 | 0.17 | 0.07 | 0.11 | 0.00 | #N/A | 0.01 | 0.32 | -0.22 | 0.00 | -0.02 | 0.16 | 0.00 | #N/A |
| Chronic renal failure [CKD] | -0.03 | 0.24 | -0.01 | 0.07 | 0.00 | 0.32 | 0.03 | 0.41 | -0.01 | 0.16 | 0.02 | 0.15 | -0.08 | 0.08 | 0.00 | 0.82 | -0.01 | 0.24 |
| Coronary atherosclerosis | 0.01 | 0.38 | 0.07 | 0.00 | 0.12 | 0.00 | 0.04 | 0.14 | 0.26 | 0.00 | 0.27 | 0.00 | 0.05 | 0.22 | -0.06 | 0.00 | -0.06 | 0.05 |
| Cough | -0.03 | 0.36 | 0.01 | 0.27 | 0.01 | 0.32 | 0.00 | 0.98 | -0.01 | 0.39 | 0.00 | #N/A | -0.04 | 0.68 | 0.01 | 0.12 | 0.01 | 0.32 |
| Degeneration of intervertebral disc | -0.02 | 0.44 | -0.01 | 0.28 | 0.00 | 0.58 | -0.08 | 0.04 | 0.00 | 0.68 | 0.00 | 0.32 | -0.13 | 0.09 | 0.00 | 0.33 | 0.02 | 0.21 |
| Depression | -0.05 | 0.03 | 0.00 | 0.40 | 0.00 | 0.88 | 0.08 | 0.13 | 0.00 | 0.36 | 0.00 | 0.32 | 0.02 | 0.81 | 0.01 | 0.28 | 0.00 | 0.32 |
| Dermatophytosis | 0.07 | 0.06 | 0.03 | 0.10 | 0.02 | 0.18 | 0.04 | 0.39 | 0.01 | 0.06 | 0.04 | 0.05 | -0.04 | 0.53 | 0.03 | 0.07 | -0.01 | 0.71 |
| Diabetic retinopathy | 0.09 | 0.03 | 0.01 | 0.13 | 0.02 | 0.28 | 0.36 | 0.00 | 0.01 | 0.04 | 0.00 | 0.32 | -0.06 | 0.70 | -0.02 | 0.25 | -0.04 | 0.06 |
| Dizziness and giddiness (Light-headedness and vertigo) | 0.06 | 0.19 | 0.00 | 0.65 | -0.01 | 0.51 | -0.02 | 0.68 | 0.00 | 0.32 | 0.01 | 0.16 | 0.05 | 0.49 | 0.04 | 0.11 | 0.00 | #N/A |
| Edema | 0.05 | 0.35 | 0.08 | 0.00 | 0.06 | 0.09 | 0.13 | 0.04 | 0.12 | 0.00 | 0.09 | 0.01 | 0.05 | 0.62 | -0.10 | 0.01 | -0.01 | 0.32 |
| Electrolyte imbalance | -0.08 | 0.09 | 0.02 | 0.11 | -0.02 | 0.09 | 0.34 | 0.00 | 0.02 | 0.17 | 0.04 | 0.04 | -0.14 | 0.03 | -0.02 | 0.27 | -0.05 | 0.32 |
| Esophagitis, GERD and related diseases | -0.04 | 0.23 | -0.01 | 0.03 | 0.00 | 0.87 | 0.06 | 0.20 | 0.00 | 0.32 | 0.00 | 0.39 | 0.05 | 0.46 | 0.02 | 0.02 | 0.02 | 0.19 |
| Essential hypertension | 0.13 | 0.00 | 0.00 | 0.16 | 0.00 | #N/A | -0.06 | 0.01 | 0.00 | 0.32 | 0.00 | #N/A | 0.17 | 0.00 | 0.00 | #N/A | 0.00 | #N/A |
| Hyperlipidemia | 0.02 | 0.49 | -0.01 | 0.16 | 0.00 | #N/A | -0.14 | 0.00 | -0.01 | 0.03 | 0.00 | 0.32 | 0.22 | 0.00 | 0.02 | 0.01 | 0.01 | 0.32 |
| Hypertensive heart and/or renal disease | 0.05 | 0.16 | 0.01 | 0.10 | 0.02 | 0.06 | -0.02 | 0.78 | 0.14 | 0.00 | 0.13 | 0.00 | 0.12 | 0.28 | -0.06 | 0.01 | -0.08 | 0.11 |
| Hypothyroidism NOS | 0.05 | 0.01 | 0.00 | 0.40 | 0.00 | 0.32 | -0.11 | 0.00 | 0.00 | 0.09 | 0.00 | 0.32 | 0.07 | 0.16 | 0.01 | 0.10 | 0.03 | 0.04 |
| Insomnia | 0.00 | 0.95 | 0.00 | 0.77 | 0.00 | 0.83 | -0.13 | 0.04 | 0.01 | 0.24 | 0.00 | #N/A | -0.03 | 0.71 | -0.01 | 0.28 | -0.01 | 0.32 |
| Muscle weakness | 0.18 | 0.00 | 0.03 | 0.01 | 0.00 | 0.15 | -0.03 | 0.50 | 0.01 | 0.14 | 0.01 | 0.25 | 0.12 | 0.13 | -0.04 | 0.02 | 0.01 | 0.39 |
| Obesity | 0.01 | 0.09 | 0.00 | 0.17 | 0.02 | 0.03 | -0.13 | 0.03 | 0.00 | 0.44 | 0.00 | 0.85 | 0.17 | 0.07 | -0.01 | 0.11 | 0.00 | 0.32 |
| Open-angle glaucoma | -0.01 | 0.83 | -0.02 | 0.01 | 0.00 | #N/A | 0.03 | 0.67 | -0.01 | 0.09 | -0.01 | 0.31 | 0.06 | 0.63 | 0.02 | 0.07 | 0.03 | 0.38 |
| Osteoarthritis; localized | -0.01 | 0.50 | -0.01 | 0.04 | 0.01 | 0.10 | -0.12 | 0.00 | 0.00 | 0.68 | 0.03 | 0.11 | 0.01 | 0.86 | 0.01 | 0.31 | -0.01 | 0.20 |
| Osteoarthrosis NOS | 0.03 | 0.61 | 0.02 | 0.02 | 0.02 | 0.08 | 0.05 | 0.53 | 0.01 | 0.14 | 0.02 | 0.13 | -0.06 | 0.35 | 0.02 | 0.53 | 0.04 | 0.32 |
| Other abnormal blood chemistry | 0.06 | 0.28 | 0.00 | 0.89 | -0.04 | 0.12 | -0.08 | 0.31 | -0.01 | 0.10 | 0.00 | #N/A | 0.08 | 0.48 | -0.03 | 0.21 | -0.03 | 0.32 |
| Other chronic nonalcoholic liver disease | 0.05 | 0.17 | 0.00 | 0.85 | 0.00 | 0.62 | 0.05 | 0.43 | 0.00 | 0.69 | 0.00 | #N/A | -0.04 | 0.65 | 0.02 | 0.42 | 0.01 | 0.32 |
| Other dyspnea | 0.12 | 0.03 | 0.04 | 0.04 | 0.04 | 0.16 | -0.04 | 0.19 | 0.02 | 0.08 | 0.02 | 0.07 | -0.01 | 0.88 | -0.02 | 0.55 | -0.04 | 0.26 |
| Peripheral vascular disease, unspecified | 0.05 | 0.47 | 0.03 | 0.02 | 0.01 | 0.70 | 0.12 | 0.08 | 0.07 | 0.00 | 0.03 | 0.12 | -0.06 | 0.31 | -0.07 | 0.00 | 0.00 | 0.70 |
| Senile cataract | -0.10 | 0.00 | -0.01 | 0.04 | -0.02 | 0.30 | 0.08 | 0.12 | -0.01 | 0.13 | 0.00 | 0.32 | 0.08 | 0.28 | 0.01 | 0.12 | 0.02 | 0.25 |
| Shortness of breath | 0.09 | 0.06 | 0.10 | 0.00 | 0.10 | 0.00 | 0.01 | 0.69 | 0.06 | 0.00 | 0.08 | 0.00 | -0.23 | 0.00 | -0.03 | 0.02 | -0.03 | 0.12 |
| Sleep apnea | -0.07 | 0.00 | 0.01 | 0.03 | 0.01 | 0.01 | -0.16 | 0.00 | 0.01 | 0.09 | 0.00 | 0.08 | 0.03 | 0.60 | 0.00 | 0.32 | 0.00 | 0.97 |
| Spondylosis without myelopathy | 0.00 | 0.89 | 0.00 | 0.78 | 0.00 | 0.97 | -0.01 | 0.86 | 0.00 | 0.88 | 0.00 | #N/A | -0.01 | 0.91 | 0.00 | 0.69 | 0.00 | #N/A |
| Type 1 diabetes | -0.03 | 0.69 | 0.02 | 0.23 | 0.08 | 0.03 | 1.05 | 0.00 | 0.01 | 0.46 | 0.01 | 0.17 | -0.72 | 0.00 | -0.02 | 0.39 | 0.00 | 0.68 |
| Type 2 diabetes | 0.03 | 0.15 | 0.00 | #N/A | 0.00 | 0.32 | 0.01 | 0.29 | -0.01 | 0.09 | 0.00 | 0.32 | 0.02 | 0.44 | 0.01 | 0.03 | 0.01 | 0.17 |
| Vitamin D deficiency | 0.05 | 0.12 | 0.00 | 0.41 | -0.01 | 0.39 | -0.29 | 0.00 | -0.01 | 0.13 | 0.00 | #N/A | 0.01 | 0.84 | 0.01 | 0.03 | -0.01 | 0.71 |
| **(Intercept)** | 0.48 | 0.00 | #N/A | #N/A | #N/A | #N/A | 0.48 | 0.00 | #N/A | #N/A | #N/A | #N/A | 0.48 | 0.00 | #N/A | #N/A | #N/A | #N/A |

### Table S3. Baseline characteristics of patients included in the study.

Values reported are Mean (SD) or N (%). N(Insulin): 195,850; N(DPP-4): 107,560. S: model-based survival probability difference; PC1, PC2, PC3: the 1^st^, 2^nd^ and 3^rd^ principal component of the comorbidities.

|  | Before IPW | | After IPW | |
| --- | --- | --- | --- | --- |
|  | Insulin | DPP4 | Insulin | DPP4 |
| **Demographics** |  |  |  |  |
| Age at First Prescription (years) | 69.45 (11.21) | 70.37 (10.98) | 69.64 (11.31) | 69.92 (10.87) |
| Disease Duration (months) | 2.4 (9.44) | 5.05 (12.34) | 2.96 (10.1) | 4.21 (11.69) |
| N (%) Male | 93539 (47.76%) | 53460 (49.7%) | 94411 (48.21%) | 52741 (49.03%) |
| N (%) Medicaid | 3997 (2.04%) | 2621 (2.44%) | 4158 (2.12%) | 2474 (2.3%) |
| Rural Status |  |  |  |  |
| N (%) Rural | 52357 (26.73%) | 25426 (23.64%) | 51186 (26.14%) | 26629 (24.76%) |
| N (%) Urban | 57527 (29.37%) | 35960 (33.43%) | 59454 (30.36%) | 34026 (31.63%) |
| Socioeconomic Index | 52 (2.94) | 52.27 (3.13) | 52.02 (2.93) | 52.23 (3.14) |
| **Laboratory** |  |  |  |  |
| A1C (%) | 8.55 (1.12) | 8.29 (1.15) | 8.51 (1.12) | 8.39 (1.18) |
| Cholesterol (mg/dL) | 170.65 (21.85) | 169.49 (24.84) | 170.24 (22.55) | 170.36 (24.15) |
| HDL (mg/dL) | 45.57 (6.27) | 45.75 (7.46) | 45.57 (6.5) | 45.72 (7.16) |
| LDL (mg/dL) | 89.88 (17.14) | 88.98 (19.93) | 89.58 (17.63) | 89.64 (19.31) |
| **Additional Medications** |  |  |  |  |
| Metformin | 0.53 (0.79) | 0.72 (0.91) | 0.6 (0.84) | 0.63 (0.86) |
| Statins | 0.71 (0.83) | 0.96 (0.92) | 0.79 (0.87) | 0.85 (0.89) |
| Sulfonylureas | 0.3 (0.66) | 0.57 (0.88) | 0.38 (0.75) | 0.46 (0.8) |
| Thiazolidinediones | 0.06 (0.32) | 0.11 (0.42) | 0.08 (0.36) | 0.09 (0.39) |
| **Others** |  |  |  |  |
| PC1 | -0.03 (2.42) | 0.03 (2.43) | 0 (2.47) | -0.01 (2.44) |
| PC2 | -0.06 (0.96) | 0.18 (1.04) | 0.02 (1) | 0.06 (1.04) |
| PC3 | 0 (0.81) | 0 (0.88) | 0.01 (0.84) | -0.02 (0.88) |
| S | -0.13 (0.08) | -0.11 (0.08) | -0.12 (0.08) | -0.12 (0.08) |

| **Disease** | Before IPW | | After IPW | |
| --- | --- | --- | --- | --- |
|  | Insulin | DPP4 | Insulin | DPP4 |
| Abnormal electrocardiogram [ECG] [EKG] | 0.07 (0.32) | 0.08 (0.35) | 0.08 (0.33) | 0.08 (0.34) |
| Abnormal glucose | 0.07 (0.33) | 0.09 (0.35) | 0.08 (0.34) | 0.08 (0.34) |
| Abnormality of gait | 0.08 (0.42) | 0.09 (0.45) | 0.08 (0.43) | 0.09 (0.44) |
| Acute renal failure | 0.1 (0.48) | 0.1 (0.49) | 0.1 (0.48) | 0.11 (0.52) |
| Anxiety disorder | 0.14 (0.53) | 0.16 (0.55) | 0.15 (0.54) | 0.15 (0.55) |
| Atrial fibrillation and flutter | 0.11 (0.52) | 0.12 (0.58) | 0.11 (0.54) | 0.12 (0.57) |
| Cerebral ischemia | 0.07 (0.37) | 0.07 (0.38) | 0.07 (0.37) | 0.07 (0.38) |
| Chronic pain | 0.08 (0.39) | 0.08 (0.39) | 0.09 (0.39) | 0.08 (0.4) |
| Chronic renal failure [CKD] | 0.28 (0.79) | 0.28 (0.79) | 0.28 (0.79) | 0.29 (0.82) |
| Coronary atherosclerosis | 0.3 (0.82) | 0.31 (0.85) | 0.31 (0.83) | 0.31 (0.85) |
| Cough | 0.12 (0.41) | 0.14 (0.45) | 0.13 (0.43) | 0.14 (0.43) |
| Degeneration of intervertebral disc | 0.08 (0.39) | 0.1 (0.44) | 0.09 (0.41) | 0.09 (0.43) |
| Depression | 0.18 (0.6) | 0.18 (0.61) | 0.19 (0.61) | 0.19 (0.62) |
| Dermatophytosis | 0.11 (0.39) | 0.12 (0.42) | 0.11 (0.4) | 0.12 (0.41) |
| Diabetic retinopathy | 0.18 (0.56) | 0.13 (0.49) | 0.17 (0.55) | 0.15 (0.54) |
| Dizziness and giddiness | 0.08 (0.36) | 0.1 (0.4) | 0.08 (0.38) | 0.09 (0.39) |
| Edema | 0.1 (0.4) | 0.1 (0.4) | 0.1 (0.4) | 0.1 (0.41) |
| Electrolyte imbalance | 0.11 (0.47) | 0.12 (0.48) | 0.12 (0.47) | 0.12 (0.5) |
| Esophagitis, GERD and related diseases | 0.22 (0.62) | 0.27 (0.67) | 0.23 (0.64) | 0.25 (0.65) |
| Essential hypertension | 1.15 (1.24) | 1.4 (1.27) | 1.23 (1.26) | 1.29 (1.26) |
| Hyperlipidemia | 0.95 (1.11) | 1.23 (1.16) | 1.03 (1.14) | 1.11 (1.14) |
| Hypertensive heart and/or renal disease | 0.21 (0.66) | 0.24 (0.69) | 0.22 (0.67) | 0.24 (0.7) |
| Hypothyroidism NOS | 0.2 (0.62) | 0.24 (0.68) | 0.21 (0.64) | 0.22 (0.66) |
| Insomnia | 0.06 (0.29) | 0.07 (0.32) | 0.06 (0.3) | 0.06 (0.31) |
| Muscle weakness | 0.09 (0.47) | 0.09 (0.47) | 0.09 (0.47) | 0.09 (0.48) |
| Obesity | 0.4 (0.85) | 0.42 (0.85) | 0.42 (0.86) | 0.41 (0.85) |
| Open-angle glaucoma | 0.06 (0.3) | 0.09 (0.37) | 0.07 (0.32) | 0.08 (0.34) |
| Osteoarthritis; localized | 0.17 (0.58) | 0.22 (0.66) | 0.19 (0.61) | 0.2 (0.64) |
| Osteoarthrosis NOS | 0.09 (0.39) | 0.1 (0.43) | 0.09 (0.4) | 0.1 (0.41) |
| Other abnormal blood chemistry | 0.06 (0.3) | 0.08 (0.34) | 0.07 (0.31) | 0.07 (0.32) |
| Other chronic nonalcoholic liver disease | 0.07 (0.36) | 0.08 (0.36) | 0.07 (0.37) | 0.07 (0.36) |
| Other dyspnea | 0.08 (0.35) | 0.09 (0.38) | 0.08 (0.36) | 0.09 (0.37) |
| Peripheral vascular disease, unspecified | 0.08 (0.38) | 0.08 (0.38) | 0.08 (0.38) | 0.08 (0.38) |
| Senile cataract | 0.14 (0.46) | 0.19 (0.52) | 0.16 (0.48) | 0.17 (0.49) |
| Shortness of breath | 0.16 (0.52) | 0.18 (0.55) | 0.17 (0.53) | 0.17 (0.55) |
| Sleep apnea | 0.2 (0.64) | 0.21 (0.67) | 0.2 (0.65) | 0.21 (0.67) |
| Spondylosis without myelopathy | 0.1 (0.44) | 0.12 (0.48) | 0.11 (0.46) | 0.11 (0.47) |
| Type 1 diabetes | 0.07 (0.31) | 0.03 (0.21) | 0.06 (0.29) | 0.06 (0.29) |
| Type 2 diabetes | 1.73 (1.44) | 1.95 (1.39) | 1.81 (1.45) | 1.85 (1.41) |
| Vitamin D deficiency | 0.18 (0.51) | 0.24 (0.59) | 0.2 (0.55) | 0.22 (0.56) |

### **Table S4.** Variables selected by PS and OR models. Each block of columns is one set of coefficients for each age spline basis. “cox1” indicates the outcome regression Cox model for insulin and “cox0” indicates the outcome regression Cox model for the comparison treatment group (DPP-4I). Coefficient estimates significantly different from zero are colored red.

|  | 1 | | | | | | 2 | | | | | | 3 | | | | | |
| --- | --- | --- | --- | --- | --- | --- | --- | --- | --- | --- | --- | --- | --- | --- | --- | --- | --- | --- |
|  | propen | propen pval | cox1 | cox1 pval | cox0 | cox0 pval | propen | propen pval | cox1 | cox1 pval | cox0 | cox0 pval | propen | propen pval | cox1 | cox1 pval | cox0 | cox0 pval |
| **Demographics** |  |  |  |  |  |  |  |  |  |  |  |  |  |  |  |  |  |  |
| Age at First Prescription (years) | -0.16 | 0.05 | 0.01 | 0.15 | 0.00 | #N/A | -0.29 | 0.04 | 0.00 | #N/A | -0.02 | 0.15 | 0.04 | 0.23 | 0.08 | 0.02 | 0.05 | 0.07 |
| Disease Duration (months) | 0.00 | 0.16 | 0.00 | 0.05 | 0.00 | 0.10 | -0.01 | 0.01 | 0.00 | 0.03 | 0.00 | 0.08 | 0.00 | #N/A | 0.00 | 0.18 | 0.00 | 0.32 |
| Male | 0.02 | 0.14 | 0.00 | 0.13 | 0.01 | 0.30 | -0.12 | 0.00 | 0.00 | 0.32 | 0.00 | 0.32 | 0.05 | 0.01 | 0.02 | 0.01 | 0.04 | 0.01 |
| Medicaid | -0.58 | 0.00 | 0.01 | 0.22 | 0.01 | 0.12 | 0.16 | 0.30 | 0.02 | 0.08 | 0.03 | 0.09 | -0.41 | 0.00 | 0.00 | 0.89 | 0.01 | 0.52 |
| Rural | 0.00 | 0.85 | 0.01 | 0.07 | 0.04 | 0.00 | 0.07 | 0.00 | 0.02 | 0.00 | 0.01 | 0.09 | -0.10 | 0.00 | -0.01 | 0.32 | 0.00 | 0.32 |
| Socioeconomic Index | 0.00 | #N/A | 0.00 | 0.32 | 0.00 | #N/A | 0.00 | #N/A | 0.00 | 0.00 | -0.01 | 0.00 | 0.00 | #N/A | 0.01 | 0.00 | 0.01 | 0.00 |
| Urban | -0.13 | 0.00 | 0.00 | 0.32 | 0.00 | #N/A | -0.22 | 0.00 | -0.02 | 0.03 | -0.01 | 0.24 | -0.15 | 0.00 | 0.02 | 0.06 | 0.02 | 0.17 |
| **Laboratory** |  |  |  |  |  |  |  |  |  |  |  |  |  |  |  |  |  |  |
| A1C | 0.07 | 0.00 | 0.00 | 0.07 | 0.00 | 0.25 | 0.26 | 0.00 | 0.00 | #N/A | 0.00 | 0.27 | 0.00 | 0.18 | 0.01 | 0.00 | 0.02 | 0.00 |
| Cholesterol | 0.00 | #N/A | 0.00 | 0.07 | 0.00 | #N/A | 0.00 | #N/A | 0.00 | 0.10 | 0.00 | 0.19 | 0.00 | #N/A | 0.00 | 0.00 | 0.00 | 0.01 |
| HDL | 0.00 | #N/A | 0.00 | #N/A | 0.00 | #N/A | 0.00 | #N/A | 0.00 | 0.01 | 0.00 | 0.01 | 0.00 | #N/A | 0.00 | 0.00 | 0.00 | 0.03 |
| LDL | 0.00 | #N/A | 0.00 | 0.23 | 0.00 | #N/A | 0.00 | #N/A | 0.00 | 0.12 | 0.00 | 0.32 | 0.00 | #N/A | 0.00 | 0.00 | 0.00 | 0.00 |
| **Additional Medications** |  |  |  |  |  |  |  |  |  |  |  |  |  |  |  |  |  |  |
| Metformin | -0.01 | 0.28 | 0.00 | 0.23 | 0.00 | 0.67 | -0.02 | 0.33 | 0.00 | 0.15 | -0.01 | 0.11 | -0.07 | 0.01 | 0.00 | 0.13 | 0.00 | #N/A |
| Statins | -0.05 | 0.02 | 0.00 | 0.32 | 0.00 | #N/A | -0.25 | 0.00 | 0.00 | #N/A | 0.00 | 0.32 | 0.02 | 0.18 | 0.00 | #N/A | 0.00 | 0.08 |
| Sulfonylureas | -0.14 | 0.00 | 0.00 | 0.15 | 0.00 | #N/A | -0.47 | 0.00 | 0.00 | 0.24 | 0.00 | 0.20 | -0.07 | 0.01 | 0.00 | 0.32 | 0.00 | 0.32 |
| Thiazolidinediones | -0.06 | 0.06 | -0.01 | 0.11 | 0.00 | 0.63 | -0.20 | 0.00 | -0.02 | 0.11 | 0.00 | #N/A | -0.16 | 0.00 | 0.01 | 0.22 | 0.01 | 0.20 |
| **Others** |  |  |  |  |  |  |  |  |  |  |  |  |  |  |  |  |  |  |
| PC1 | -0.01 | 0.66 | 0.00 | 0.29 | 0.00 | #N/A | -0.01 | 0.68 | 0.00 | #N/A | 0.00 | #N/A | -0.01 | 0.37 | 0.00 | 0.46 | -0.01 | 0.14 |
| PC2 | 0.00 | 0.32 | -0.02 | 0.00 | -0.02 | 0.01 | -0.32 | 0.00 | -0.57 | 0.00 | -0.60 | 0.00 | 0.00 | 0.92 | 0.01 | 0.05 | 0.01 | 0.02 |
| PC3 | -0.01 | 0.10 | 0.00 | 0.96 | 0.00 | 0.83 | -0.02 | 0.16 | 0.00 | 0.75 | 0.00 | 0.42 | 0.00 | 0.32 | 0.00 | 0.74 | 0.00 | 0.41 |
| S | -0.61 | 0.01 | -1.42 | 0.00 | -0.11 | 0.09 | -0.20 | 0.11 | -6.83 | 0.00 | 1.63 | 0.00 | 0.22 | 0.05 | -0.21 | 0.01 | -0.06 | 0.29 |
| **Disease** |  |  |  |  |  |  |  |  |  |  |  |  |  |  |  |  |  |  |
| Abnormal electrocardiogram [ECG] [EKG] | -0.08 | 0.00 | 0.01 | 0.03 | 0.00 | 0.32 | 0.00 | 0.99 | 0.01 | 0.07 | 0.00 | 0.38 | -0.06 | 0.31 | -0.01 | 0.07 | -0.01 | 0.21 |
| Abnormal glucose | -0.05 | 0.11 | 0.00 | 0.09 | -0.01 | 0.20 | -0.04 | 0.36 | -0.01 | 0.07 | -0.02 | 0.15 | 0.10 | 0.02 | 0.01 | 0.40 | 0.01 | 0.68 |
| Abnormality of gait | 0.04 | 0.22 | 0.01 | 0.10 | 0.02 | 0.02 | -0.04 | 0.33 | 0.01 | 0.23 | 0.01 | 0.25 | -0.04 | 0.07 | -0.01 | 0.21 | -0.02 | 0.22 |
| Acute renal failure | -0.03 | 0.21 | 0.00 | 0.74 | 0.01 | 0.18 | 0.02 | 0.47 | 0.00 | 0.39 | 0.02 | 0.02 | -0.01 | 0.70 | -0.01 | 0.35 | -0.01 | 0.23 |
| Anxiety disorder | -0.04 | 0.05 | 0.00 | 0.32 | 0.00 | 0.54 | 0.01 | 0.68 | 0.00 | 0.32 | 0.00 | 0.32 | 0.02 | 0.59 | 0.00 | 0.32 | 0.00 | 0.38 |
| Atrial fibrillation and flutter | 0.00 | 0.77 | 0.04 | 0.00 | 0.05 | 0.00 | -0.01 | 0.58 | 0.33 | 0.00 | 0.33 | 0.00 | 0.00 | 0.81 | 0.01 | 0.09 | 0.01 | 0.12 |
| Cerebral ischemia | -0.05 | 0.23 | 0.01 | 0.10 | 0.03 | 0.01 | 0.16 | 0.00 | 0.01 | 0.10 | 0.02 | 0.04 | -0.05 | 0.21 | -0.02 | 0.05 | 0.00 | 0.78 |
| Chronic pain | 0.04 | 0.29 | 0.00 | 0.32 | 0.01 | 0.09 | 0.09 | 0.03 | -0.01 | 0.22 | 0.00 | 0.31 | -0.03 | 0.68 | -0.01 | 0.21 | 0.00 | 0.83 |
| Chronic renal failure [CKD] | 0.00 | 0.99 | 0.00 | 0.30 | 0.00 | 0.32 | 0.05 | 0.02 | 0.00 | 0.17 | 0.00 | 0.32 | -0.01 | 0.64 | 0.00 | 0.54 | 0.00 | 0.20 |
| Coronary atherosclerosis | 0.00 | 0.76 | 0.04 | 0.00 | 0.05 | 0.00 | 0.06 | 0.00 | 0.25 | 0.00 | 0.25 | 0.00 | -0.02 | 0.36 | -0.01 | 0.05 | 0.00 | 0.52 |
| Cough | -0.03 | 0.07 | 0.00 | 0.81 | 0.01 | 0.12 | -0.02 | 0.51 | 0.00 | 0.32 | 0.00 | 0.32 | 0.02 | 0.36 | 0.01 | 0.04 | 0.00 | 0.33 |
| Degeneration of intervertebral disc | 0.01 | 0.83 | -0.01 | 0.15 | 0.01 | 0.17 | -0.04 | 0.14 | 0.00 | 0.38 | -0.01 | 0.10 | 0.00 | 0.99 | 0.00 | 0.99 | 0.01 | 0.37 |
| Depression | 0.01 | 0.51 | 0.00 | 0.42 | 0.00 | 0.16 | 0.05 | 0.03 | 0.00 | 0.07 | 0.00 | 0.56 | 0.03 | 0.28 | 0.01 | 0.10 | 0.01 | 0.32 |
| Dermatophytosis | 0.02 | 0.47 | 0.00 | 0.16 | 0.00 | 0.21 | 0.02 | 0.46 | 0.01 | 0.22 | 0.01 | 0.16 | 0.02 | 0.63 | 0.00 | 0.32 | 0.01 | 0.24 |
| Diabetic retinopathy | 0.09 | 0.00 | 0.00 | 0.13 | 0.01 | 0.12 | 0.40 | 0.00 | 0.00 | 0.10 | 0.01 | 0.25 | 0.01 | 0.77 | 0.00 | 0.94 | -0.01 | 0.17 |
| Dizziness and giddiness (Light-headedness and vertigo) | -0.02 | 0.39 | 0.00 | 0.25 | 0.00 | 0.83 | -0.01 | 0.77 | -0.01 | 0.10 | 0.00 | 0.99 | -0.04 | 0.39 | 0.00 | 0.71 | 0.01 | 0.74 |
| Edema | 0.02 | 0.38 | 0.06 | 0.00 | 0.05 | 0.00 | 0.08 | 0.00 | 0.17 | 0.00 | 0.17 | 0.00 | -0.08 | 0.02 | -0.02 | 0.03 | -0.02 | 0.26 |
| Electrolyte imbalance | -0.07 | 0.00 | 0.01 | 0.32 | 0.01 | 0.07 | 0.07 | 0.03 | 0.00 | 0.76 | 0.00 | 0.12 | -0.09 | 0.00 | -0.01 | 0.48 | -0.01 | 0.64 |
| Esophagitis, GERD and related diseases | -0.04 | 0.06 | -0.01 | 0.11 | 0.00 | 0.08 | -0.07 | 0.00 | -0.01 | 0.14 | 0.00 | #N/A | -0.02 | 0.59 | 0.00 | 0.74 | 0.00 | #N/A |
| Essential hypertension | -0.07 | 0.00 | 0.00 | 0.32 | 0.00 | 0.32 | -0.06 | 0.01 | 0.00 | 0.16 | 0.00 | #N/A | -0.02 | 0.20 | 0.00 | #N/A | 0.00 | 0.32 |
| Hyperlipidemia | -0.09 | 0.00 | 0.00 | 0.09 | 0.00 | 0.19 | -0.14 | 0.00 | -0.02 | 0.01 | -0.01 | 0.02 | -0.02 | 0.27 | 0.00 | 0.32 | 0.00 | 0.19 |
| Hypertensive heart and/or renal disease | -0.06 | 0.00 | 0.01 | 0.03 | 0.01 | 0.06 | -0.12 | 0.00 | 0.08 | 0.00 | 0.07 | 0.00 | -0.02 | 0.43 | -0.03 | 0.02 | -0.02 | 0.07 |
| Hypothyroidism NOS | 0.02 | 0.25 | 0.00 | 0.32 | 0.00 | 0.45 | -0.04 | 0.08 | -0.01 | 0.02 | 0.00 | #N/A | -0.04 | 0.05 | 0.00 | 0.28 | 0.00 | 1.00 |
| Insomnia | -0.02 | 0.45 | 0.00 | 0.21 | 0.00 | 0.65 | 0.00 | 0.95 | 0.00 | 0.65 | 0.00 | 0.24 | -0.01 | 0.87 | -0.01 | 0.23 | 0.02 | 0.19 |
| Muscle weakness | 0.04 | 0.11 | 0.02 | 0.00 | 0.00 | 0.32 | 0.05 | 0.11 | 0.01 | 0.10 | 0.01 | 0.09 | 0.03 | 0.21 | 0.00 | 0.39 | 0.00 | 0.55 |
| Obesity | 0.02 | 0.10 | 0.00 | 0.05 | 0.01 | 0.12 | 0.06 | 0.00 | 0.00 | #N/A | 0.00 | #N/A | 0.00 | 0.99 | 0.00 | 0.25 | 0.00 | 0.32 |
| Open-angle glaucoma | -0.10 | 0.01 | -0.01 | 0.14 | 0.00 | 0.32 | -0.10 | 0.01 | -0.01 | 0.10 | -0.01 | 0.12 | -0.02 | 0.63 | 0.00 | 0.38 | 0.00 | 0.32 |
| Osteoarthritis; localized | -0.05 | 0.01 | 0.00 | 0.32 | 0.00 | #N/A | -0.04 | 0.04 | 0.00 | 0.92 | 0.00 | 0.20 | 0.02 | 0.58 | 0.01 | 0.18 | 0.00 | 0.89 |
| Osteoarthrosis NOS | -0.04 | 0.14 | 0.00 | 0.94 | -0.01 | 0.35 | -0.02 | 0.57 | 0.00 | 0.70 | 0.00 | #N/A | -0.03 | 0.38 | 0.00 | 0.83 | -0.01 | 0.24 |
| Other abnormal blood chemistry | -0.02 | 0.47 | 0.00 | 0.32 | 0.00 | 0.47 | -0.08 | 0.02 | 0.00 | 0.32 | 0.00 | 0.32 | 0.01 | 0.90 | 0.00 | 0.77 | 0.01 | 0.41 |
| Other chronic nonalcoholic liver disease | 0.01 | 0.82 | -0.01 | 0.17 | 0.00 | 0.96 | -0.01 | 0.83 | 0.00 | 0.24 | 0.00 | #N/A | -0.11 | 0.06 | 0.00 | 0.32 | 0.00 | 0.32 |
| Other dyspnea | 0.01 | 0.59 | 0.01 | 0.16 | 0.02 | 0.02 | -0.03 | 0.37 | 0.03 | 0.01 | 0.04 | 0.00 | 0.00 | 0.99 | 0.00 | 0.72 | -0.01 | 0.44 |
| Peripheral vascular disease, unspecified | 0.04 | 0.29 | 0.02 | 0.01 | 0.03 | 0.01 | 0.09 | 0.02 | 0.04 | 0.00 | 0.03 | 0.03 | 0.00 | 0.94 | -0.02 | 0.02 | -0.01 | 0.28 |
| Senile cataract | -0.13 | 0.00 | -0.01 | 0.11 | -0.01 | 0.17 | -0.03 | 0.21 | 0.00 | 0.32 | -0.01 | 0.12 | -0.06 | 0.06 | 0.00 | 0.32 | 0.00 | 0.97 |
| Shortness of breath | -0.02 | 0.28 | 0.08 | 0.00 | 0.07 | 0.00 | -0.02 | 0.47 | 0.14 | 0.00 | 0.17 | 0.00 | -0.04 | 0.41 | -0.01 | 0.02 | 0.00 | 0.25 |
| Sleep apnea | 0.04 | 0.02 | 0.00 | 0.17 | 0.00 | 0.04 | -0.02 | 0.05 | 0.02 | 0.02 | 0.01 | 0.29 | -0.02 | 0.24 | 0.00 | 0.70 | 0.01 | 0.28 |
| Spondylosis without myelopathy | -0.02 | 0.30 | 0.00 | 0.79 | 0.00 | 0.29 | -0.01 | 0.66 | 0.00 | 0.33 | 0.00 | 0.15 | -0.04 | 0.28 | 0.00 | 0.32 | 0.01 | 0.30 |
| Type 1 diabetes | 0.38 | 0.00 | 0.05 | 0.01 | 0.05 | 0.08 | 1.46 | 0.00 | 0.00 | 0.99 | 0.01 | 0.32 | 0.05 | 0.63 | 0.00 | 0.87 | 0.00 | 0.32 |
| Type 2 diabetes | 0.01 | 0.39 | 0.00 | #N/A | 0.00 | #N/A | 0.07 | 0.00 | -0.01 | 0.01 | 0.00 | 0.16 | -0.01 | 0.58 | 0.00 | 0.32 | 0.00 | #N/A |
| Vitamin D deficiency | -0.02 | 0.18 | 0.00 | 0.81 | 0.00 | 0.32 | -0.11 | 0.00 | -0.01 | 0.01 | -0.01 | 0.14 | 0.06 | 0.03 | 0.00 | 0.32 | 0.01 | 0.10 |
| **(Intercept)** | 0.02 | 0.84 | #N/A | #N/A | #N/A | #N/A | 0.02 | 0.84 | #N/A | #N/A | #N/A | #N/A | 0.02 | 0.84 | #N/A | #N/A | #N/A | #N/A |

### Figure S2. HF rates before and after IPW adjusting for insulin vs. DPP-4I comparison.

**
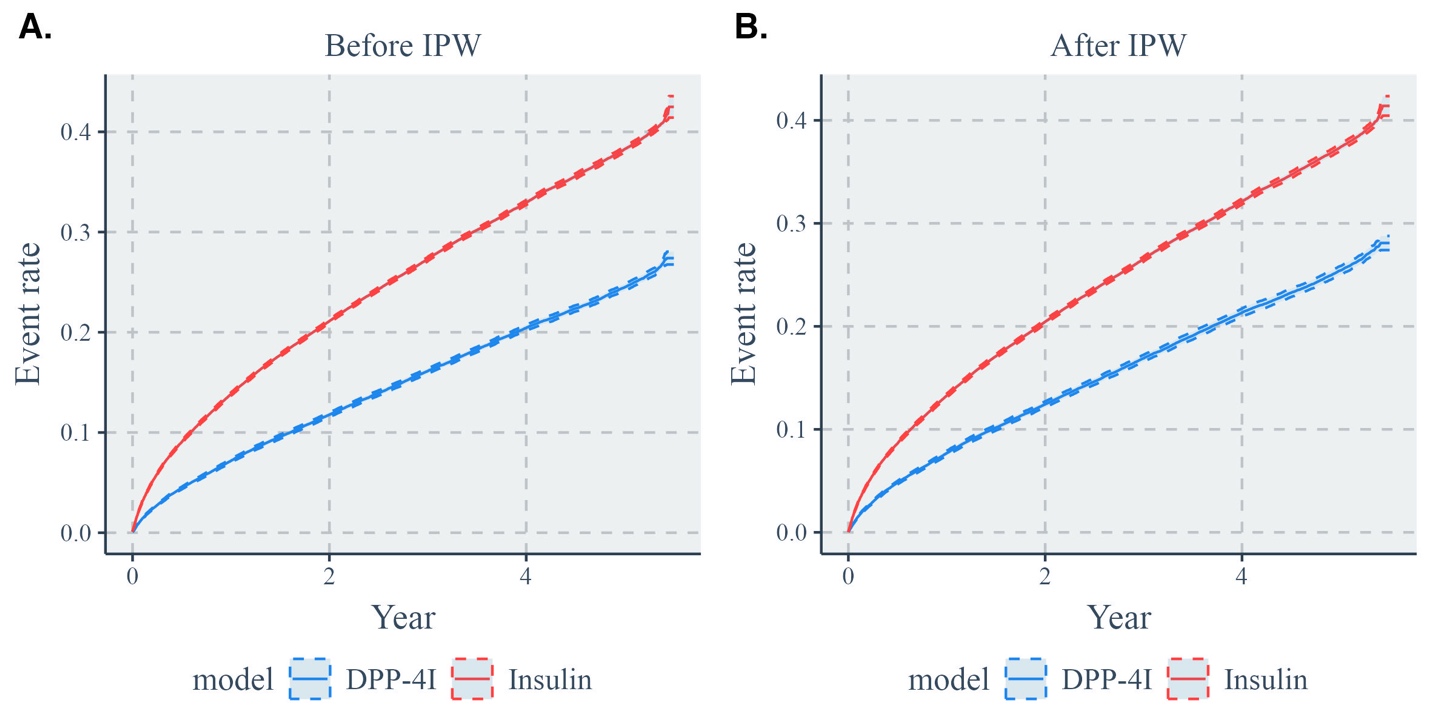
**

### Figure S3. CATE estimates for insulin vs. DPP-4I comparison.

(S: model-based survival probability difference)

**
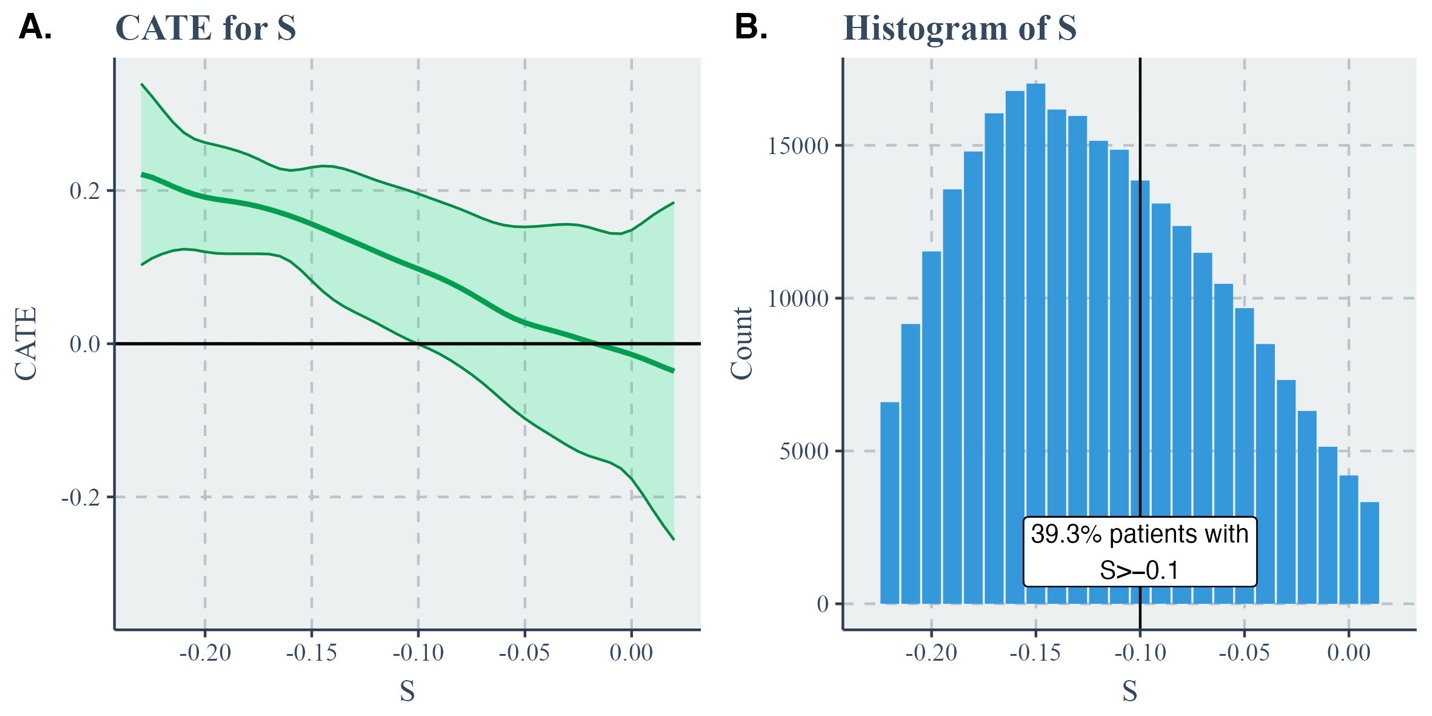
**

### Table S5. Baseline characteristics of patients included in the study.

Values reported are Mean (SD) or N (%). N(Insulin): 138,827; N(SGLT-2): 48,266. S: model-based survival probability difference; PC1, PC2, PC3: the 1^st^, 2^nd^ and 3^rd^ principal component of the comorbidities.

|  | Before IPW | | After IPW | |
| --- | --- | --- | --- | --- |
|  | Insulin | SGLT2 | Insulin | SGLT2 |
| **Demographics** |  |  |  |  |
| Age at First Prescription (years) | 66.03 (9.89) | 63.14 (10.49) | 65.26 (10.27) | 65.01 (9.91) |
| Disease Duration (months) | 2.76 (9.92) | 8.34 (14.85) | 3.73 (11.11) | 5.92 (13.41) |
| N (%) Male | 71276 (51.34%) | 28828 (59.73%) | 73061 (52.63%) | 27188 (56.33%) |
| N (%) Medicaid | 1802 (1.3%) | 474 (0.98%) | 1741 (1.25%) | 587 (1.22%) |
| Rural Status |  |  |  |  |
| N (%) Rural | 36882 (26.57%) | 11161 (23.12%) | 35949 (25.9%) | 12067 (25%) |
| N (%) Urban | 40528 (29.19%) | 15069 (31.22%) | 41284 (29.74%) | 14345 (29.72%) |
| Socioeconomic Index | 52.03 (2.94) | 52.37 (3.03) | 52.06 (2.94) | 52.29 (3.03) |
| **Laboratory** |  |  |  |  |
| A1C (%) | 8.54 (1.12) | 8.34 (1.23) | 8.55 (1.17) | 8.38 (1.19) |
| Cholesterol (mg/dL) | 170.85 (22.37) | 170.69 (27.99) | 170.71 (23.52) | 171.01 (25.95) |
| HDL (mg/dL) | 45.47 (6.32) | 45.21 (8.03) | 45.36 (6.64) | 45.49 (7.5) |
| LDL (mg/dL) | 90.04 (17.51) | 89.69 (22.26) | 89.92 (18.39) | 89.92 (20.59) |
| **Additional Medications** |  |  |  |  |
| Metformin | 0.61 (0.83) | 1.02 (0.95) | 0.72 (0.89) | 0.79 (0.88) |
| Statins | 0.73 (0.83) | 1.08 (0.95) | 0.81 (0.88) | 0.89 (0.89) |
| Sulfonylureas | 0.32 (0.68) | 0.56 (0.89) | 0.38 (0.75) | 0.44 (0.79) |
| Thiazolidinediones | 0.07 (0.34) | 0.14 (0.48) | 0.09 (0.38) | 0.11 (0.41) |
| **Others** |  |  |  |  |
| PC1 | 0 (2.21) | 0.01 (2.14) | -0.01 (2.27) | 0.01 (2.17) |
| PC2 | 0.16 (0.75) | 0.55 (0.85) | 0.25 (0.81) | 0.31 (0.79) |
| PC3 | 0 (0.73) | -0.01 (0.86) | -0.01 (0.76) | 0 (0.82) |
| S | -0.17 (0.09) | -0.13 (0.09) | -0.16 (0.1) | -0.16 (0.09) |

| **Disease** | Before IPW | | After IPW | |
| --- | --- | --- | --- | --- |
|  | Insulin | SGLT2 | Insulin | SGLT2 |
| Abnormal electrocardiogram [ECG] [EKG] | 0.05 (0.28) | 0.07 (0.33) | 0.06 (0.29) | 0.07 (0.32) |
| Abnormal glucose | 0.06 (0.31) | 0.1 (0.38) | 0.07 (0.33) | 0.08 (0.35) |
| Abnormality of gait | 0.04 (0.28) | 0.04 (0.3) | 0.04 (0.29) | 0.04 (0.3) |
| Acute renal failure | 0.04 (0.27) | 0.03 (0.26) | 0.04 (0.27) | 0.04 (0.28) |
| Anxiety disorder | 0.12 (0.47) | 0.16 (0.55) | 0.14 (0.5) | 0.15 (0.52) |
| Atrial fibrillation and flutter | 0.07 (0.4) | 0.08 (0.45) | 0.07 (0.41) | 0.08 (0.45) |
| Cerebral ischemia | 0.04 (0.27) | 0.04 (0.29) | 0.04 (0.28) | 0.04 (0.28) |
| Chronic pain | 0.06 (0.33) | 0.07 (0.35) | 0.07 (0.34) | 0.07 (0.35) |
| Chronic renal failure [CKD] | 0.11 (0.44) | 0.08 (0.4) | 0.11 (0.43) | 0.1 (0.44) |
| Coronary atherosclerosis | 0.23 (0.71) | 0.29 (0.8) | 0.24 (0.73) | 0.28 (0.79) |
| Cough | 0.1 (0.38) | 0.13 (0.43) | 0.11 (0.4) | 0.12 (0.42) |
| Degeneration of intervertebral disc | 0.07 (0.36) | 0.09 (0.42) | 0.08 (0.38) | 0.09 (0.41) |
| Depression | 0.15 (0.52) | 0.17 (0.57) | 0.16 (0.54) | 0.16 (0.55) |
| Dermatophytosis | 0.08 (0.33) | 0.09 (0.37) | 0.08 (0.34) | 0.09 (0.35) |
| Diabetic retinopathy | 0.14 (0.48) | 0.11 (0.46) | 0.14 (0.48) | 0.12 (0.48) |
| Dizziness and giddiness | 0.06 (0.32) | 0.08 (0.37) | 0.07 (0.33) | 0.07 (0.35) |
| Edema | 0.06 (0.3) | 0.06 (0.32) | 0.06 (0.31) | 0.07 (0.32) |
| Electrolyte imbalance | 0.06 (0.33) | 0.06 (0.33) | 0.06 (0.33) | 0.06 (0.33) |
| Esophagitis, GERD and related diseases | 0.19 (0.56) | 0.24 (0.63) | 0.2 (0.58) | 0.22 (0.6) |
| Essential hypertension | 1.09 (1.18) | 1.41 (1.22) | 1.18 (1.21) | 1.24 (1.2) |
| Hyperlipidemia | 0.94 (1.08) | 1.35 (1.13) | 1.05 (1.11) | 1.13 (1.11) |
| Hypertensive heart and/or renal disease | 0.11 (0.43) | 0.11 (0.45) | 0.11 (0.43) | 0.12 (0.46) |
| Hypothyroidism NOS | 0.18 (0.57) | 0.23 (0.66) | 0.19 (0.6) | 0.2 (0.62) |
| Insomnia | 0.05 (0.27) | 0.07 (0.32) | 0.05 (0.28) | 0.06 (0.3) |
| Muscle weakness | 0.04 (0.3) | 0.04 (0.32) | 0.04 (0.3) | 0.04 (0.32) |
| Obesity | 0.39 (0.81) | 0.52 (0.9) | 0.43 (0.85) | 0.46 (0.86) |
| Open-angle glaucoma | 0.05 (0.28) | 0.07 (0.34) | 0.06 (0.3) | 0.06 (0.31) |
| Osteoarthritis; localized | 0.15 (0.53) | 0.2 (0.62) | 0.16 (0.55) | 0.18 (0.59) |
| Osteoarthrosis NOS | 0.06 (0.31) | 0.07 (0.34) | 0.06 (0.32) | 0.07 (0.34) |
| Other abnormal blood chemistry | 0.05 (0.26) | 0.07 (0.32) | 0.05 (0.28) | 0.06 (0.29) |
| Other chronic nonalcoholic liver disease | 0.06 (0.34) | 0.09 (0.39) | 0.07 (0.36) | 0.08 (0.36) |
| Other dyspnea | 0.06 (0.3) | 0.07 (0.34) | 0.06 (0.31) | 0.07 (0.33) |
| Peripheral vascular disease, unspecified | 0.05 (0.3) | 0.05 (0.31) | 0.05 (0.3) | 0.06 (0.31) |
| Senile cataract | 0.14 (0.44) | 0.18 (0.5) | 0.15 (0.46) | 0.16 (0.48) |
| Shortness of breath | 0.12 (0.43) | 0.13 (0.47) | 0.12 (0.44) | 0.13 (0.47) |
| Sleep apnea | 0.18 (0.6) | 0.24 (0.71) | 0.19 (0.63) | 0.22 (0.67) |
| Spondylosis without myelopathy | 0.09 (0.41) | 0.11 (0.47) | 0.1 (0.43) | 0.1 (0.45) |
| Type 1 diabetes | 0.03 (0.2) | 0.02 (0.17) | 0.03 (0.2) | 0.03 (0.2) |
| Type 2 diabetes | 1.66 (1.36) | 2.09 (1.24) | 1.78 (1.37) | 1.85 (1.3) |
| Vitamin D deficiency | 0.17 (0.5) | 0.26 (0.63) | 0.19 (0.54) | 0.21 (0.56) |

### **Table S6.** Variables selected by PS and OR models. Each block of columns is one set of coefficients for each age spline basis. “cox1” indicates the outcome regression Cox model for insulin and “cox0” indicates the outcome regression Cox model for the comparison treatment group (SGLT-2). Coefficient estimates significantly different from zero are colored red.

|  | 1 | | | | | | 2 | | | | | | 3 | | | | | |
| --- | --- | --- | --- | --- | --- | --- | --- | --- | --- | --- | --- | --- | --- | --- | --- | --- | --- | --- |
|  | propen | propen pval | cox1 | cox1 pval | cox0 | cox0 pval | propen | propen pval | cox1 | cox1 pval | cox0 | cox0 pval | propen | propen pval | cox1 | cox1 pval | cox0 | cox0 pval |
| **Demographics** |  |  |  |  |  |  |  |  |  |  |  |  |  |  |  |  |  |  |
| Age at First Prescription (years) | 0.04 | 0.21 | 0.02 | 0.07 | 0.01 | 0.32 | -0.13 | 0.15 | 0.00 | #N/A | 0.00 | #N/A | 0.14 | 0.06 | 0.00 | #N/A | 0.03 | 0.11 |
| Disease Duration (months) | 0.00 | 0.18 | 0.00 | 0.18 | 0.00 | #N/A | -0.03 | 0.00 | 0.00 | 0.25 | 0.00 | #N/A | 0.00 | #N/A | 0.00 | 0.32 | 0.00 | #N/A |
| Male | 0.01 | 0.80 | 0.04 | 0.00 | 0.08 | 0.01 | -0.34 | 0.00 | 0.00 | #N/A | 0.00 | 0.32 | 0.21 | 0.00 | 0.01 | 0.10 | 0.00 | #N/A |
| Medicaid | -0.43 | 0.00 | 0.16 | 0.01 | 0.20 | 0.03 | 0.79 | 0.00 | 0.05 | 0.01 | 0.06 | 0.16 | -0.51 | 0.04 | -0.13 | 0.20 | -0.13 | 0.08 |
| Rural | 0.00 | 0.87 | 0.02 | 0.01 | 0.04 | 0.04 | 0.13 | 0.00 | 0.03 | 0.01 | -0.01 | 0.32 | -0.10 | 0.13 | -0.02 | 0.11 | 0.00 | #N/A |
| Socioeconomic Index | 0.00 | #N/A | 0.00 | #N/A | 0.00 | 0.32 | 0.00 | #N/A | 0.00 | 0.07 | 0.00 | 0.32 | 0.00 | 0.32 | 0.00 | 0.00 | 0.00 | 0.00 |
| Urban | 0.10 | 0.01 | 0.00 | 0.84 | 0.03 | 0.32 | -0.08 | 0.20 | -0.01 | 0.04 | 0.00 | #N/A | 0.01 | 0.90 | 0.08 | 0.00 | 0.05 | 0.09 |
| **Laboratory** |  |  |  |  |  |  |  |  |  |  |  |  |  |  |  |  |  |  |
| A1C | 0.03 | 0.00 | 0.01 | 0.00 | 0.00 | 0.07 | 0.19 | 0.00 | 0.00 | #N/A | 0.00 | #N/A | -0.03 | 0.23 | 0.00 | 0.09 | 0.00 | 0.19 |
| Cholesterol | 0.00 | #N/A | 0.00 | 0.02 | 0.00 | 0.16 | 0.00 | #N/A | 0.00 | #N/A | 0.00 | 0.32 | 0.00 | #N/A | 0.00 | 0.14 | 0.00 | 0.10 |
| HDL | 0.00 | #N/A | 0.00 | 0.32 | 0.00 | #N/A | 0.00 | #N/A | 0.00 | 0.32 | 0.00 | #N/A | 0.00 | #N/A | 0.00 | 0.02 | 0.00 | 0.03 |
| LDL | 0.00 | #N/A | 0.00 | 0.12 | 0.00 | 0.05 | 0.00 | #N/A | 0.00 | 0.18 | 0.00 | 0.32 | 0.00 | #N/A | 0.00 | 0.07 | 0.00 | 0.15 |
| **Additional Medications** |  |  |  |  |  |  |  |  |  |  |  |  |  |  |  |  |  |  |
| Metformin | -0.02 | 0.11 | 0.00 | #N/A | 0.00 | #N/A | -0.05 | 0.03 | 0.00 | #N/A | 0.00 | #N/A | 0.11 | 0.01 | 0.01 | 0.13 | 0.01 | 0.12 |
| Statins | 0.00 | 0.89 | 0.00 | 0.19 | 0.01 | 0.23 | -0.07 | 0.04 | 0.00 | 0.32 | 0.00 | #N/A | 0.15 | 0.02 | 0.00 | 0.95 | 0.00 | #N/A |
| Sulfonylureas | -0.05 | 0.02 | 0.00 | 0.17 | 0.00 | 0.24 | -0.04 | 0.03 | 0.00 | #N/A | -0.01 | 0.32 | 0.09 | 0.01 | -0.01 | 0.41 | 0.00 | #N/A |
| Thiazolidinediones | -0.08 | 0.04 | 0.00 | 0.56 | -0.01 | 0.32 | -0.13 | 0.05 | -0.01 | 0.02 | 0.00 | 0.32 | 0.10 | 0.36 | 0.04 | 0.04 | 0.02 | 0.32 |
| **Others** |  |  |  |  |  |  |  |  |  |  |  |  |  |  |  |  |  |  |
| PC1 | 0.03 | 0.29 | 0.00 | 0.40 | 0.00 | #N/A | -0.02 | 0.36 | 0.00 | #N/A | 0.00 | #N/A | -0.02 | 0.82 | 0.00 | #N/A | 0.00 | #N/A |
| PC2 | -0.01 | 0.58 | -0.03 | 0.00 | 0.00 | 0.32 | -0.63 | 0.00 | -0.44 | 0.00 | -0.18 | 0.00 | 0.05 | 0.15 | 0.12 | 0.00 | 0.11 | 0.01 |
| PC3 | 0.01 | 0.56 | 0.01 | 0.33 | 0.00 | #N/A | 0.05 | 0.11 | 0.00 | #N/A | 0.00 | #N/A | -0.03 | 0.23 | 0.00 | 0.90 | 0.00 | 0.32 |
| S | -4.68 | 0.00 | -1.82 | 0.00 | -0.28 | 0.02 | -0.74 | 0.05 | -5.79 | 0.00 | 0.76 | 0.05 | -0.16 | 0.58 | -0.08 | 0.14 | -0.36 | 0.09 |
| **Disease** |  |  |  |  |  |  |  |  |  |  |  |  |  |  |  |  |  |  |
| Abnormal electrocardiogram [ECG] [EKG] | 0.03 | 0.49 | 0.00 | 0.93 | 0.05 | 0.22 | -0.11 | 0.16 | 0.01 | 0.12 | 0.01 | 0.22 | -0.04 | 0.69 | -0.01 | 0.65 | -0.02 | 0.32 |
| Abnormal glucose | -0.08 | 0.10 | 0.00 | 0.70 | 0.00 | 0.32 | 0.00 | 0.98 | 0.00 | 0.18 | 0.00 | 0.32 | 0.27 | 0.02 | 0.04 | 0.09 | 0.00 | #N/A |
| Abnormality of gait | 0.13 | 0.03 | 0.02 | 0.05 | 0.04 | 0.06 | -0.04 | 0.18 | 0.02 | 0.07 | 0.00 | #N/A | -0.14 | 0.11 | 0.00 | 0.86 | 0.02 | 0.44 |
| Acute renal failure | -0.05 | 0.22 | 0.05 | 0.02 | 0.05 | 0.03 | 0.23 | 0.00 | 0.04 | 0.11 | 0.05 | 0.03 | -0.19 | 0.09 | -0.07 | 0.17 | -0.02 | 0.81 |
| Anxiety disorder | 0.00 | 0.97 | 0.00 | 0.32 | 0.01 | 0.41 | -0.03 | 0.53 | -0.02 | 0.12 | 0.00 | 0.32 | -0.03 | 0.70 | 0.01 | 0.38 | -0.03 | 0.32 |
| Atrial fibrillation and flutter | -0.01 | 0.87 | 0.13 | 0.00 | 0.14 | 0.00 | -0.11 | 0.03 | 0.28 | 0.00 | 0.28 | 0.00 | -0.11 | 0.08 | -0.06 | 0.01 | -0.07 | 0.04 |
| Cerebral ischemia | 0.00 | 0.97 | 0.02 | 0.22 | 0.02 | 0.21 | 0.06 | 0.32 | 0.02 | 0.10 | 0.06 | 0.04 | -0.21 | 0.03 | -0.02 | 0.18 | 0.04 | 0.15 |
| Chronic pain | -0.07 | 0.07 | 0.00 | 0.34 | 0.01 | 0.16 | 0.17 | 0.00 | 0.00 | 0.32 | 0.03 | 0.26 | -0.28 | 0.00 | -0.04 | 0.09 | -0.01 | 0.16 |
| Chronic renal failure [CKD] | 0.04 | 0.08 | -0.02 | 0.06 | 0.01 | 0.17 | 0.35 | 0.00 | -0.01 | 0.19 | 0.02 | 0.21 | -0.09 | 0.23 | 0.01 | 0.65 | 0.00 | 0.92 |
| Coronary atherosclerosis | -0.05 | 0.01 | 0.05 | 0.00 | 0.08 | 0.00 | -0.06 | 0.05 | 0.22 | 0.00 | 0.24 | 0.00 | 0.05 | 0.24 | -0.04 | 0.04 | -0.06 | 0.03 |
| Cough | -0.03 | 0.53 | 0.01 | 0.21 | 0.05 | 0.05 | 0.11 | 0.11 | 0.00 | #N/A | 0.00 | #N/A | 0.05 | 0.62 | -0.01 | 0.49 | 0.00 | #N/A |
| Degeneration of intervertebral disc | -0.06 | 0.20 | 0.01 | 0.19 | 0.02 | 0.28 | 0.13 | 0.08 | 0.00 | #N/A | 0.01 | 0.25 | 0.19 | 0.06 | 0.02 | 0.16 | -0.03 | 0.23 |
| Depression | -0.01 | 0.86 | 0.01 | 0.39 | 0.01 | 0.17 | 0.05 | 0.15 | 0.00 | 0.42 | 0.00 | #N/A | -0.05 | 0.04 | 0.01 | 0.19 | -0.04 | 0.27 |
| Dermatophytosis | -0.02 | 0.69 | 0.02 | 0.10 | 0.00 | 0.32 | 0.10 | 0.02 | 0.00 | 0.17 | 0.02 | 0.09 | -0.13 | 0.04 | -0.01 | 0.16 | 0.00 | #N/A |
| Diabetic retinopathy | 0.06 | 0.02 | 0.00 | 0.74 | 0.01 | 0.20 | 0.28 | 0.00 | 0.00 | 0.22 | 0.01 | 0.32 | -0.23 | 0.03 | -0.01 | 0.32 | 0.01 | 0.32 |
| Dizziness and giddiness (Light-headedness and vertigo) | 0.03 | 0.63 | 0.01 | 0.07 | 0.01 | 0.23 | -0.05 | 0.30 | 0.00 | 0.23 | 0.00 | #N/A | -0.08 | 0.23 | 0.02 | 0.08 | 0.03 | 0.18 |
| Edema | 0.07 | 0.29 | 0.08 | 0.00 | 0.16 | 0.00 | 0.10 | 0.17 | 0.08 | 0.00 | 0.07 | 0.02 | -0.22 | 0.01 | -0.10 | 0.02 | -0.02 | 0.23 |
| Electrolyte imbalance | 0.00 | 0.97 | 0.04 | 0.11 | 0.07 | 0.04 | 0.26 | 0.00 | 0.01 | 0.20 | 0.03 | 0.16 | -0.16 | 0.02 | -0.02 | 0.38 | 0.00 | 0.82 |
| Esophagitis, GERD and related diseases | -0.03 | 0.20 | -0.01 | 0.11 | 0.00 | #N/A | -0.03 | 0.36 | 0.00 | 0.32 | 0.00 | #N/A | 0.04 | 0.54 | 0.02 | 0.06 | 0.00 | 0.62 |
| Essential hypertension | 0.04 | 0.04 | 0.00 | 0.32 | 0.00 | #N/A | -0.02 | 0.59 | 0.00 | 0.32 | 0.00 | #N/A | 0.05 | 0.25 | 0.00 | #N/A | 0.00 | #N/A |
| Hyperlipidemia | 0.01 | 0.15 | 0.00 | 0.14 | 0.00 | #N/A | -0.15 | 0.00 | -0.01 | 0.10 | 0.00 | 0.32 | 0.16 | 0.00 | 0.03 | 0.05 | 0.01 | 0.23 |
| Hypertensive heart and/or renal disease | 0.02 | 0.64 | 0.03 | 0.04 | 0.07 | 0.00 | -0.11 | 0.09 | 0.25 | 0.00 | 0.29 | 0.00 | 0.04 | 0.50 | -0.08 | 0.00 | -0.05 | 0.11 |
| Hypothyroidism NOS | 0.00 | 0.68 | 0.00 | 0.49 | 0.01 | 0.30 | -0.04 | 0.34 | 0.00 | 0.30 | 0.00 | 0.32 | -0.01 | 0.92 | 0.00 | 0.30 | 0.00 | 0.92 |
| Insomnia | 0.09 | 0.07 | 0.00 | 0.76 | 0.00 | 0.32 | -0.17 | 0.00 | 0.00 | 0.32 | 0.00 | #N/A | -0.09 | 0.24 | 0.02 | 0.53 | 0.00 | #N/A |
| Muscle weakness | 0.09 | 0.09 | 0.04 | 0.07 | 0.04 | 0.08 | 0.07 | 0.31 | 0.01 | 0.05 | 0.02 | 0.25 | 0.04 | 0.67 | -0.03 | 0.10 | 0.02 | 0.31 |
| Obesity | 0.00 | 0.86 | 0.01 | 0.05 | 0.01 | 0.13 | -0.02 | 0.51 | 0.00 | 0.35 | 0.00 | 0.32 | 0.04 | 0.35 | 0.00 | #N/A | 0.00 | #N/A |
| Open-angle glaucoma | -0.08 | 0.20 | 0.00 | 0.32 | 0.00 | #N/A | 0.07 | 0.41 | -0.01 | 0.12 | 0.00 | #N/A | 0.19 | 0.07 | 0.02 | 0.27 | 0.02 | 0.20 |
| Osteoarthritis; localized | -0.04 | 0.15 | -0.01 | 0.07 | -0.01 | 0.10 | 0.00 | 1.00 | -0.01 | 0.18 | -0.01 | 0.47 | 0.03 | 0.66 | 0.01 | 0.16 | 0.01 | 0.32 |
| Osteoarthrosis NOS | 0.02 | 0.71 | 0.02 | 0.11 | 0.01 | 0.53 | 0.11 | 0.03 | 0.01 | 0.26 | 0.01 | 0.18 | 0.02 | 0.81 | 0.03 | 0.32 | 0.03 | 0.21 |
| Other abnormal blood chemistry | 0.05 | 0.26 | -0.01 | 0.39 | 0.02 | 0.20 | -0.15 | 0.01 | -0.01 | 0.17 | 0.00 | #N/A | -0.10 | 0.24 | 0.00 | 0.32 | 0.00 | #N/A |
| Other chronic nonalcoholic liver disease | 0.05 | 0.30 | 0.00 | 0.51 | 0.04 | 0.05 | 0.02 | 0.29 | 0.00 | 0.41 | 0.00 | #N/A | -0.01 | 0.59 | 0.01 | 0.44 | 0.00 | #N/A |
| Other dyspnea | 0.03 | 0.51 | 0.03 | 0.04 | 0.03 | 0.16 | -0.09 | 0.06 | 0.03 | 0.02 | 0.03 | 0.15 | -0.14 | 0.02 | -0.04 | 0.23 | 0.00 | 0.94 |
| Peripheral vascular disease, unspecified | 0.08 | 0.10 | 0.05 | 0.01 | 0.08 | 0.01 | 0.00 | 0.95 | 0.10 | 0.00 | 0.07 | 0.01 | -0.20 | 0.00 | -0.07 | 0.02 | -0.11 | 0.07 |
| Senile cataract | -0.08 | 0.01 | 0.00 | 0.18 | 0.00 | #N/A | 0.00 | 0.98 | -0.01 | 0.20 | 0.00 | #N/A | 0.02 | 0.71 | 0.00 | 0.32 | 0.00 | 0.32 |
| Shortness of breath | 0.04 | 0.22 | 0.12 | 0.00 | 0.10 | 0.00 | 0.09 | 0.07 | 0.03 | 0.00 | 0.08 | 0.01 | 0.08 | 0.27 | -0.02 | 0.13 | 0.00 | 0.32 |
| Sleep apnea | -0.02 | 0.25 | 0.02 | 0.02 | 0.03 | 0.03 | -0.04 | 0.01 | 0.02 | 0.01 | 0.00 | 0.17 | 0.06 | 0.26 | 0.00 | 0.32 | -0.02 | 0.15 |
| Spondylosis without myelopathy | -0.02 | 0.44 | 0.01 | 0.19 | 0.01 | 0.22 | -0.07 | 0.12 | -0.01 | 0.10 | 0.00 | #N/A | -0.25 | 0.01 | 0.00 | 0.32 | 0.00 | #N/A |
| Type 1 diabetes | 0.11 | 0.34 | 0.07 | 0.03 | 0.07 | 0.03 | 1.25 | 0.00 | 0.04 | 0.08 | 0.00 | #N/A | -0.49 | 0.05 | -0.03 | 0.19 | -0.07 | 0.14 |
| Type 2 diabetes | 0.05 | 0.01 | 0.00 | #N/A | 0.00 | #N/A | 0.01 | 0.26 | -0.01 | 0.02 | 0.00 | 0.32 | 0.04 | 0.31 | 0.02 | 0.01 | 0.00 | 0.16 |
| Vitamin D deficiency | -0.01 | 0.82 | -0.01 | 0.45 | 0.00 | #N/A | -0.13 | 0.00 | -0.01 | 0.09 | 0.00 | #N/A | 0.07 | 0.18 | 0.02 | 0.05 | 0.02 | 0.32 |
| **(Intercept)** | 0.56 | 0.00 | #N/A | #N/A | #N/A | #N/A | 0.56 | 0.00 | #N/A | #N/A | #N/A | #N/A | 0.56 | 0.00 | #N/A | #N/A | #N/A | #N/A |

### Figure S4. Survival curves before and after IPW adjusting for insulin vs. SGLT-2 comparison

**
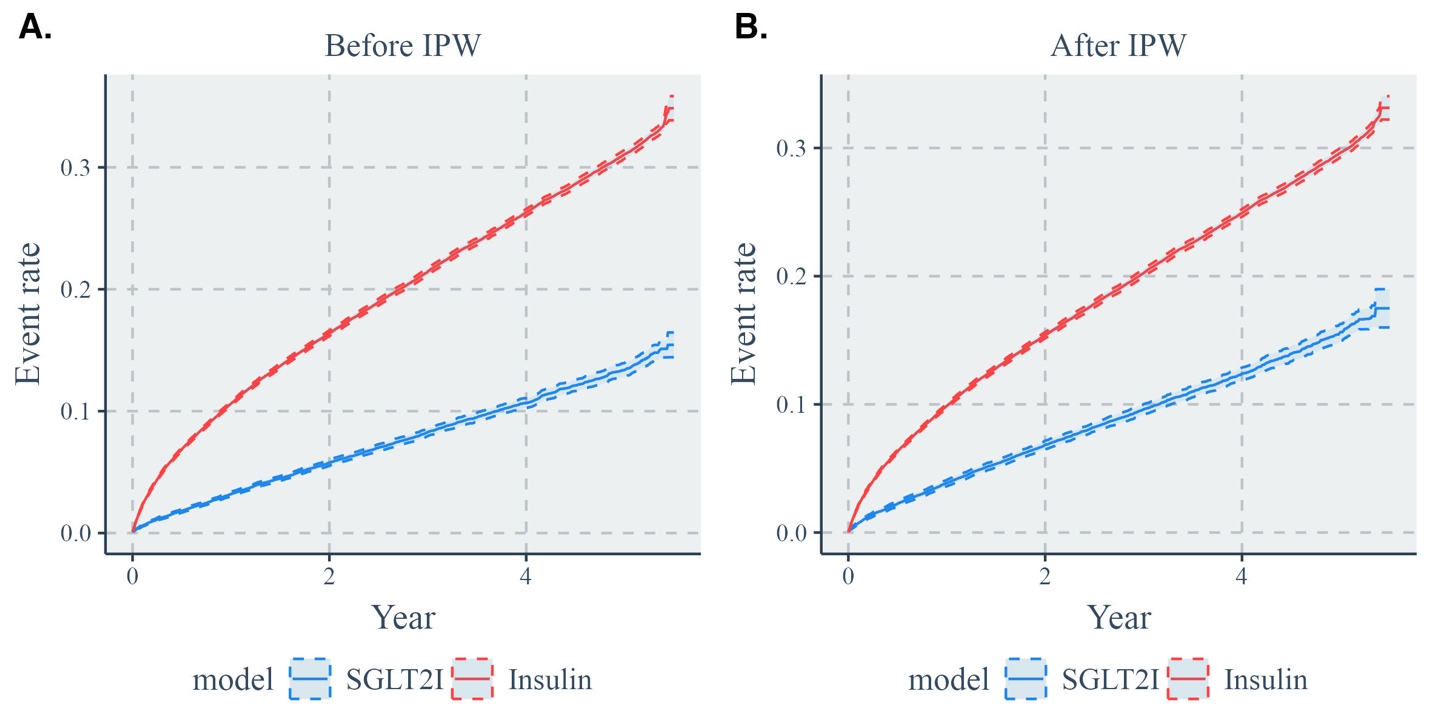
**

### Figure S5. CATE estimates for insulin vs. SGLT-2 comparison.

(S: model-based survival probability difference)


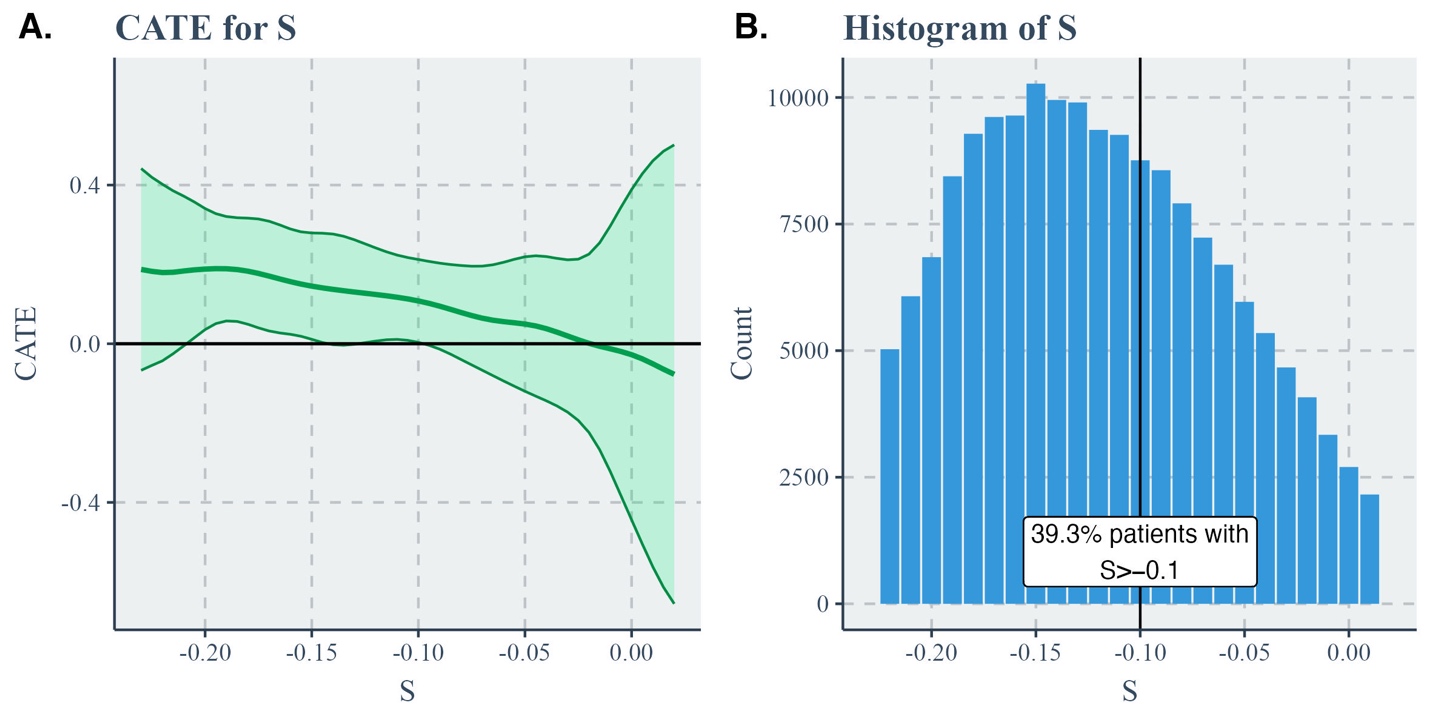

Supplement: Multimedia Appendix 1 [file diabetes-v9-e58137-s001.docx]
